# Sphingolipid-dependent Dscam sorting regulates axon segregation

**DOI:** 10.1038/s41467-019-08765-2

**Published:** 2019-02-18

**Authors:** Gaurav Goyal, Junfeng Zheng, Elisabeth Adam, Georg Steffes, Mamta Jain, Kristaps Klavins, Thomas Hummel

**Affiliations:** 1 Department for Neurobiology, University of Vienna, Althanstrasse 14, A-1090 Vienna, Austria; 2 Institut für Neuro- und Verhaltensbiologie, Universität Münster, Badestrasse 9/13, D-48149 Münster, Germany; 3 Division of Cell and Developmental Biology, Centre for Anatomy and Cell Biology, Medical University of Vienna, Schwarzspanierstrasse 17, A-1090 Vienna, Austria; 4 CeMM Research Centre for Molecular Medicine of the Austrian Academy of Sciences, Lazarettgasse 14, AKH, A-1090 Vienna, Austria; 5 Present Address: Medical Department, Xaimen University, Xaimen, 361005 China

## Abstract

Neurons are highly polarized cells with distinct protein compositions in axonal and dendritic compartments. Cellular mechanisms controlling polarized protein sorting have been described for mature nervous system but little is known about the segregation in newly differentiated neurons. In a forward genetic screen for regulators of *Drosophila* brain circuit development, we identified mutations in SPT, an evolutionary conserved enzyme in sphingolipid biosynthesis. Here we show that reduced levels of sphingolipids in *SPT* mutants cause axonal morphology defects similar to loss of cell recognition molecule Dscam. Loss- and gain-of-function studies show that neuronal sphingolipids are critical to prevent aggregation of axonal and dendritic Dscam isoforms, thereby ensuring precise Dscam localization to support axon branch segregation. Furthermore, *SPT* mutations causing neurodegenerative HSAN-I disorder in humans also result in formation of stable Dscam aggregates and axonal branch phenotypes in *Drosophila* neurons, indicating a causal link between developmental protein sorting defects and neuronal dysfunction.

## Abstract (web summary)

Little is known about the initial segregation of axonal and dendritic proteins during the differentiation of newly generated neurons. Here authors use a forward genetic screen to identify the role of sphingolipids in regulating the sub-cellular distribution of Dscam for neuronal patterning in *Drosophila* Mushroom Bodies

## Introduction

Neurons are highly polarized cells with morphologically and functionally specialized axonal and dendritic compartments. This functional polarity is maintained by having a strict control on intra-cellular transport of vesicles carrying cargo destined for different neuronal compartments^1–3^. Although substantial progress has been achieved in the understanding of compartment-specific protein sorting in the mature nervous system^1–5^, we still have little insights into the developmental mechanisms controlling initial segregation of axonal and dendritic proteins involved in neuronal patterning^5^.

In addition to proteins, lipids define a major component of transport vesicles. Sphingolipids, typified by the presence of the long chain amino-alcohol sphingosine, are enriched in certain cellular membranes and are a major constituent of lipid rafts, specialized signaling centers in the plasma membrane^6–8^. Additionally, sphingolipids can regulate the segregation of cargos for polarized intra-cellular transport at the trans-golgi network^9^. In cultured hippocampal neurons, chemical inhibition of sphingolipid biosynthesis affects axonal outgrowth and transport of axonally targeted proteins^10,11^. However, in vivo analysis for the role of sphingolipids in polarized transport and their role in neuronal patterning and survival is largely unexplored.

The Down syndrome cell adhesion molecule (Dscam) regulates early developmental patterning of dendrites and axons in *Drosophila*^12^. Cell-intrinsic function of Dscam has been implicated in self avoidance of growing axons and dendrites and for the proper development of axonal connectivity^13–19^. These neurite patterning activities are tightly associated with the specific intra-neuronal distribution of Dscam isoforms which differ in the transmembrane domains TM1 and TM2^20–22^. Dscam[TM1] isoforms are localized to dendrites and control dendritic patterning^13,20–22^, whereas axon branching depends on Dscam[TM2] isoforms^17–23^. Thus, Dscam is a unique molecule whose cell-intrinsic function in neurite patterning depends on differential intra-cellular distribution.

Here we describe a novel role of sphingolipids in regulating the subcellular distribution of Dscam in *Drosophila* Mushroom Body (MB) neurons. The reduction in sphingolipids interferes with the initial segregation of dendritic and axonal Dscam isoforms thereby resulting in Dscam-associated neuronal patterning defects. Furthermore, the disruption of Dscam sorting is associated with the formation of stable protein aggregates, which translocate into the axonal compartment, suggesting related pathological mechanisms in human neurological disorders associated with a perturbed sphingolipid biosynthesis^24,25^.

## Results

### Loss of *SPT* leads to *Dscam*-mutant phenotypes

**Fig. 1:**
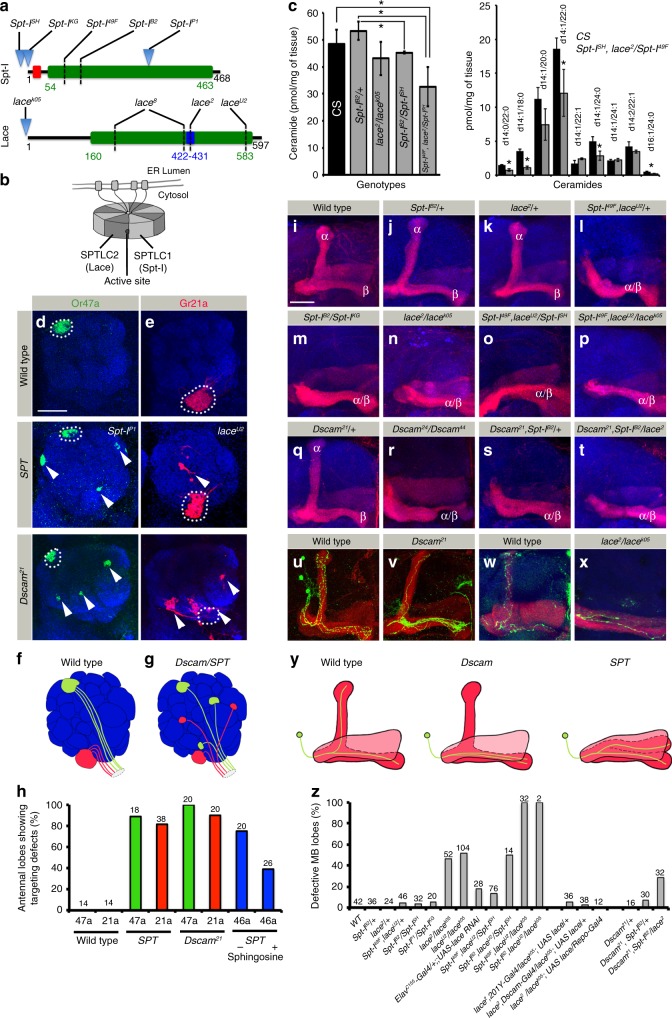
Loss of SPT leads to Dscam-like phenotypes in neuronal development. **a** Protein domain organization of the two SPT subunits of *Drosophila*, indicating the mutations used in the study. Green: amino-transferase domain, Red: N-terminal transmembrane domain in Spt-I, and Blue: a PLP binding site in Lace. Mutations indicated: *Spt-I*^*SH*^ *(Spt-I*^*SH1626*^*):* P-element insertion in 5’ UTR; *Spt-I*^*KG*^ *(Spt-I*^*KG06406*^*):* P-element insertion at 1^st^ base of SPT-I; *Spt-I*^*B2*^: G127E; *Spt-I*^*49F*^ *(Spt-I* ^*l(2)49Fb4*^*):* Q90 stop; *Spt-I*^*P1*^*:* P-element at Glu295; *lace*^*2*^ *(lace*^*HG34*^*):* S429N*; lace*^*8*^ *(lace*^*VT2*^*):* Y221S and K414Q*; lace*^*U2*^*:* C570T*; lace*^*k05*^ *(lace*^*k05305*^*)*: P-element insertion 8–10 bp upstream of transcription start site. **b** Schematic showing the octameric organization of SPT holozyme^26, 28^. Black Dot: Active site. **c** In adult flies, MS analysis showed that 5 out of 9 identified ceramide species have significantly lower levels in *SPT* mutants as compared to *CS* (Right panel), leading to significantly reduced total Ceramide levels (Left panel). Bars represent mean + /− SD across 3 biological replicates. Raw data in Supplementary Data 1. Two sided *T*-Test *P value < 0.05. **d**–**h** Homozygous mutant clones of *Spt-I*^*P1*^, *lace*^*U2*^ and *Dscam*^*21*^ show axonal mistargeting defect (arrowheads) of ORN classes Or47a ((**d**), green) and Gr21a ((**e**), red), summarized in the schematics (**f**, **g**), and quantified in **h**. The wild-type targeting site is marked with dotted circle. **h** In addition, mistargeting of Or46a (blue bars) in *lace*^*2*^*/lace*^*k05*^ is rescued following sphingosine supplementation. **i**–**t** Adult MB lobe morphology in Wild type (control) and heterozygous *Spt-I* and *lace* mutants show normal α/β lobe segregation (**i**–**k**, **z**) whereas double/trans-heterozygous mutants show defective MB axonal morphology (**l**–**p**, **z**). *Dscam* and *SPT* mutants display synergistic effect on MB lobe development (**q**–**t**). **u**–**y** MARCM clones (Green) of wild type (**u**) and *Dscam*^*21*^ mutant (**v**) MB neurons show non-segregated axon branches. Single neuron labeled in wild type (**w**) and *lace* trans-heterozygous (**x**, *lace*^*2*^*/lace*^*k05*^) background using flybow/flip out cassette. **y** Schematic showing the axonal phenotype in *Dscam* and *SPT* mutants in MB of *Drosophila*. **z** Quantification of MB lobe defects in different genetic backgrounds. **i**–**x** Red: FasII (strongly labels α/β lobes and faintly γ lobe). **d**, **e**, **i**–**x** Blue: N-Cad (neuropil marker). **h**, **z** Numbers on the bars represent number of OL/MB analyzed. Scale Bar: 25 μm

In a mosaic screen for identifying genes involved in Dscam-mediated neuronal patterning, we identified mutations in the two subunits of Serine Palmitoyltransferase (SPT), the key enzyme of de novo sphingolipid biosynthesis, encoded by the genes *Spt-I* and *lace*^26^ (Fig. 1a). The mammalian homologs SPTLC1 and SPTLC2 assemble into a large protein multimer, which localizes to the ER membrane^27,28^ (Fig. 1b). In *Drosophila*, hypomorphic *lace* mutants show a strong reduction in sphingolipid levels accompanied by enhanced cell death in imaginal discs and defective glial development^29–33^. The newly identified *Spt-I*^*B2*^ allele carries a point mutation (G127E) in the amino-transferase (AT) domain. Similarly, a single point mutation could be identified in *lace*^*U2*^ (C570T), which also maps to the predicted AT domain^26^ (Fig. 1a). In genetic complementation analyses, *Spt-I*^*B2*^ and *lace*^*U2*^ classified as strong hypomorphic mutations (Supplementary Figure 1A), suggesting a severe reduction or loss of protein function. Consistent with this, *Spt-I* and *lace* trans-heterozygotes showed lower levels of total ceramide as compared to control, further reduced in *Spt-I* and *lace* double mutant combination but no change in membrane phospholipid Phosphatidylcholine (PC) (Fig. 1c, Supplementary Figure 1B, Supplementary Data 1).

In the *Drosophila* olfactory system, olfactory receptor neurons (ORNs) homozygous mutant for *Spt-I* fail to reach their target glomerulus in the brain but converge ectopically, which is similar to *Dscam*-mutant ORNs (Fig. 1d–h). In the central brain, developing MB neurons require SPT function to segregate axonal branches into distinct lobes. This results in *SPT* mutants showing a “lost-lobe” phenotype indistinguishable from *Dscam*-mutant MB defects^15^ (Fig. 1i–t, z). Mutations in regulatory subunit *Spt-I* shows lower penetrance but identical axonal phenotypes as compared to the enzymatic subunit *lace*^26^ in the olfactory system and MB (Fig. 1d–h, i–p, z). Single cell analysis revealed a failure of *lace* mutant MB neurons to extend their axon branches dorsally but instead develop two parallel horizontal branches which has been described before following the loss of Dscam^15^ (Fig. 1u-y). A pan-neuronal reduction of sphingolipids via *lace*^*RNAi*^ showed the same but less frequent MB defects, confirming SPT function in nervous system development (Fig. 1z). Removing a copy of *Spt-I* in hypomorphic *lace* mutants (or vice versa) increases the penetrance and expressivity of MB axonal defects, indicating a direct functional interaction between the two SPT subunits in neuronal patterning (Fig. 1i–p, z). In addition, various allelic combinations showed strong genetic interactions between *SPT* and *Dscam* in MB and ORN targeting (e.g., *Dscam, Spt-I/* *+* and *Dscam, Spt-I/lace;* Fig. 1q-t, z and Supplementary Figure 2). The *lace* mutant neuronal defects can be rescued by the expression of a wild-type transgene not only in developing neurons (*Dscam-Gal4*) but also in glia cells (*repo-Gal4)*, suggesting a non-cell autonomous supply of sphingolipids^34^ (Fig. 1z). This is supported by a lack of phenotype in small *SPT* MARCM clones, showing that a loss of SPT function in developing neurons can be compensated by surrounding wild-type cells (Supplementary Figure 3). Knockdown of other enzymes catalyzing various steps of sphingolipid biosynthesis pathway also show MB lost-lobe phenotype, indicating lack of sphingolipids but not of a specific enzyme as the main factor leading to developmental defects in the nervous system (Supplementary Figure 4). This is further confirmed by the complementation of SPT function by sphingosine food supplementation leading to a significant reduction of neuronal defects in *SPT* mutants (Fig. 1h).

These data show that mutations in each of the SPT subunits result in a *Dscam-*like phenotype in the *Drosophila* nervous system. The synergistic interaction of Spt-I and Lace in early neuronal development is supporting the proposed functions of the two SPT subunits in the regulation of sphingolipid synthesis^26^. In addition, the rescue of *SPT* mutant neurons by surrounding wild-type tissue indicates a direct effect of sphingolipid levels in the brain.

### Neuron class-specific role of sphingolipids in MB development

**Fig. 2:**
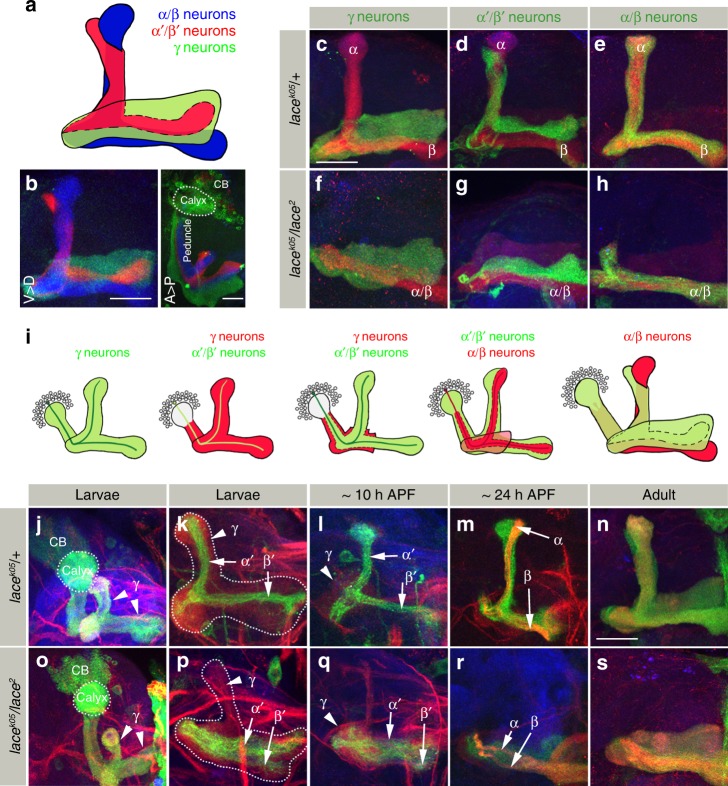
Neuron class-specific role of sphingolipids in MB development. **a** Schematic showing the three classes of MB neurons. Green: γ, Red: α′/β′ and Blue: α/β. **b** Anterior View (Ventral (V) > Dorsal (D)) and Dorsal view (Anterior (A) > Posterior (P)) of the three differently labeled classes of MB neurons. γ: mCD8::GFP, α′/β′: mCherry, α/β: anti-FasII. Genotype: *R26E01-LexA/LexAop myr::cherry; R16A06-Gal4/UAS-mCD8::GFP*. *R26E01-lexA* (α′/β′ neurons), *R16A06-Gal4* (γ neurons). **c**–**h** Morphology of MB in *lace*^*k05*^*/* *+* (**c**, **d**, **e**) and *lace*^*2*^*/lace*^*k05*^ (**f**, **g**, **h**) class specifically labeled with mCD8::GFP for γ (**c**, **f**), α′/β′ (**d**, **g**), and α/β (**e**, **h**) neurons. α′/β′ and α/β neurons show a loss of the vertical axonal lobe in *SPT* mutants. *n*(**c**) = 26, *n*(**d**) = 26, *n*(**e**) = 38, *n*(**f**) = 42, *n*(**g**) = 36, *n*(**h**) = 36. **i**–**s** Schematic showing the development of different MB neuron classes (**i**). MB morphology in *lace*^*k05*^*/* *+* (**j**–**n**) and *lace*^*2*^*/lace*^*k05*^ (**o**–**s**) at indicated developmental stages. Initially, γ neurons develop normally in *SPT* mutants ((**j**, **o**) Green). The axonal morphology defects first appear in α′/β′ neurons ((**k**, **p**) and (**l**, **q**) Green: α′/β′, Red: γ) followed by α/β neurons ((**m**, **r**) Green: α′/β′, Strong Red (FasII): α/β). MB are labeled with mCD8::GFP expressed using different Gal4s: *201Y-* ((**j**, **o**) γ neurons), *R30F11-* ((**k**–**m** and **p**–**r**) α′/β′ and early born α/β) and *OK107-Gal4* ((**n**, **s**) all classes of MB). *n*(**j**) = 38, *n*(**k**) = 12, *n*(**l**) = 6, *n*(**m**) = 16, *n*(**n**) = 48, *n*(**o**) = 30, *n*(**p**) = 26, *n*(**q**) = 8, *n*(**r**) = 10, *n*(**s**) = 52. **c**–**s** Green: GFP, Red: FasII, Blue: N-Cad. Scale Bar: 25 μm

As *Drosophila* MB consist of three main neuronal subtypes of Kenyon cells, α/β, α′/β′ and γ neurons, which organizes their axons into distinct lobe neuropils^35^ (Fig. 2a, b), we next analyzed neuron class-specific defects in *SPT* mutants. The α′/β′ neurons develop before the α/β neurons and axons of both classes tightly associate in the lobe region (Fig. 2d). The differential labeling of both MB classes in *SPT* mutants showed a strict co-occurrence of the α/α′ lobe defect supporting a previously described inter-dependency of α branches on the earlier formed α′ branches during Dscam-mediated axon pattering^15^ (Fig. 2d, g). Although co-projecting into the same direction, neuronal identity is not changed in *SPT* mutants, as α′/β′ and α/β axons still segregate into distinct horizontal lobes (Fig. 2d, e, g, h). In contrast, γ neurons, which do not form a vertical axon lobe in the adult brain, seem not affected in *SPT* mutants (Fig. 2c, f).

Next, we determined the developmental onset of MB defects in *SPT* mutants. In wild type, MB neuron subtypes are generated in a fixed developmental sequence (Fig. 2i, j–n). In accordance with the adult phenotype, in *SPT* mutants the formation of larval γ neurons is unaffected, segregating axon branches into two distinct lobes (Fig. 2o, p). In contrast, α′/β′ neurons, extending their axons at 3^rd^ instar stage (visualized by the neuronal cell surface molecule Flamingo or the Gal4 expression line *R30F11*) fail to segregate their vertical axon branch in *SPT* mutants resulting in two parallel horizontal axon fibers (Supplementary Figure 5 for anti-Flamingo staining and Fig. 2p, q). Similarly, α/β neurons do not project an axon branch vertically in *SPT* mutant pupae, leading to the “lost-lobe phenotype” in adults (Fig. 2r,s). Interestingly, initial axon extension into the proximal MB peduncle, known to require Dscam function^22^, is not affected in *SPT* mutants demonstrating a tight functional correlation between Dscam and sphingolipids for branch segregation at the distal axonal region (Fig. 2, Supplementary Figure 5).

### Sphingolipid-dependent Dscam localization

**Fig. 3:**
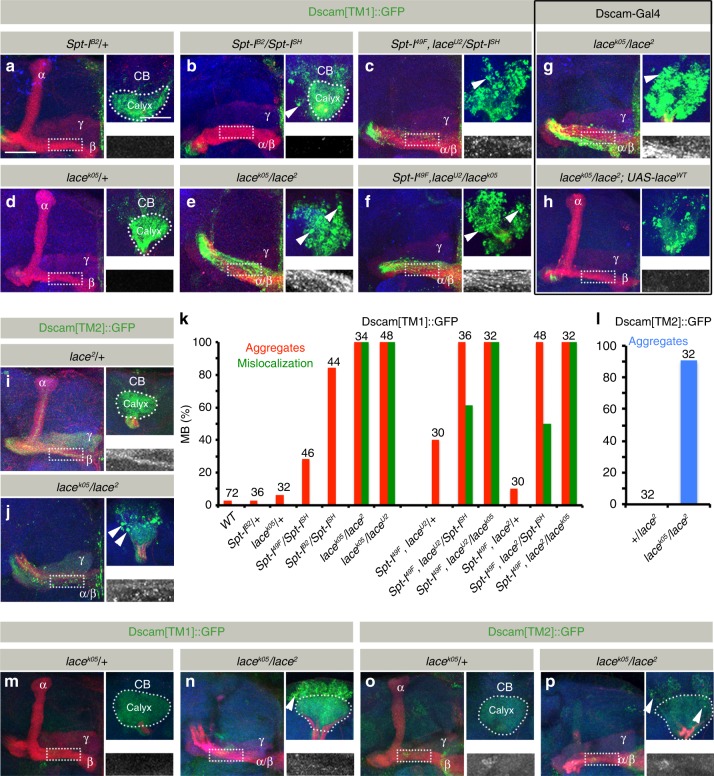
*SPT* mutants affect intra-cellular Dscam localization. **a**–**f** Pan-kenyon cell (*OK107-Gal4)* expression of Dscam[TM1]::GFP shows formation of aggregates in *SPT* mutants. While images **a**–**f** represent the axonal half of the MB, the corresponding somato-dendritic area of the same MB is represented in right upper insets. The white dotted rectangle represents the Dscam[TM1]::GFP distribution in the axons, zoomed in and shown in the right lower inset. While *Spt-I* trans-heterozygotes primarily show formation of Dscam[TM1]::GFP aggregates (**b**, **k**), an additional *lace* heterozygous mutation enhances the phenotype and leads to mislocalization of Dscam[TM1]::GFP into the axons (**c**, **k**). *lace* trans-heterozygous mutants are inherently stronger than *Spt-I* mutants and show a high penetrance of Dscam[TM1]::GFP aggregates and axonal mislocalization (**e**, **f**, **k**). **g**, **h** Like *OK107-Gal4*, expression of Dscam[TM1]::GFP using *Dscam-Gal4* shows formation of somatic aggregates and axonal mislocalization in *lace* trans-heterozygous mutants (**g**), which can be rescued by co-expressing a lace wild-type transgene (**h**). *n*(**g**) = 34, *n*(**h**) = 30. **i**, **j** Expression of Dscam[TM2]::GFP using *201Y-Gal4* also shows formation of somatic aggregates in *lace* trans-heterozygous background (**j**, **l**). **k** Percentage of MB showing somatic aggregates and axonal mislocalization of Dscam[TM1]::GFP. **l** Percentage of MB showing somatic aggregates of Dscam[TM2]::GFP. Numbers on the bars represent number of MB analyzed. **m**–**p** Dscam[TM1::GFP] (**m**, **n**) and Dscam[TM2::GFP] (**o**, **p**) expressed downsteam of Dscam regulatory region shows formation of somatic aggregates (in addition to axonal mislocalization of Dscam[TM1]) in *SPT* mutants (**n**,**p**) as compared to control (**m**, **o**). *n*(**m**) = 16, *n*(**n**) = 18, *n*(**o**) = 16, *n*(**p**) = 30. **a**–**j**, **m**–**p** Green: GFP, Red: FasII, Blue: N-Cad. Arrowheads indicate somatic aggregates of Dscam. Scale Bar: 25 μm

As the morphological changes in *SPT* mutant neurons were similar to *Dscam*, we decided to analyze the distribution of two transmembrane Dscam isoforms in *SPT* mutants. In wild type neurons, Dscam shows a distinct intra-cellular localization, depending on two alternative trans-/juxta-membrane domains:^20–22^ Dscam[TM1] localizes to the dendritic compartment, the MB calyx (Fig. 3a), whereas Dscam[TM2] is distributed throughout the neuronal membrane and enriched in axons (Fig. 3i, also see Figure Dscam[TM1] interferes with Dscam[TM2] function in SPT mutant MB neurons panels d, e).

In *SPT* mutants, this compartment-specific Dscam[TM1]/[TM2] sorting is severely affected. In mutant brains, dendritic Dscam[TM1] strongly accumulates in neuronal cell bodies of MBs. In addition, a mislocalization of dendritic Dscam[TM1] isoform to the axonal compartment, enriching in the MB lobes, can be observed (Fig. 3a–f, k). The neuronal expression of a wild type *SPT* transgene using a *Dscam-Gal4* driver line rescues the Dscam[TM1] mislocalization phenotype (Fig. 3g, h). Similar to dendritic Dscam[TM1] in *SPT* mutants, axonal Dscam[TM2] accumulates in the somato-dendritic and axonal compartments, accompanied by a reduction of Dscam[TM2] in extending axons (Fig. 3i, j, l). Different *Spt-I/lace* allelic combinations showed a direct correlation in the phenotypic strength of axonal segregation and Dscam distribution defects (Fig. 1z and Fig. 3a–f, k). Dscam distribution defects were also observed in SPT mutants on expressing Dscam[TM1] and Dscam[TM2] downstream of Dscam regulatory region^21^, indicating that the observed defects reflect distribution of endogenous Dscam isoforms (Fig. 3m–p).

**Fig. 4:**
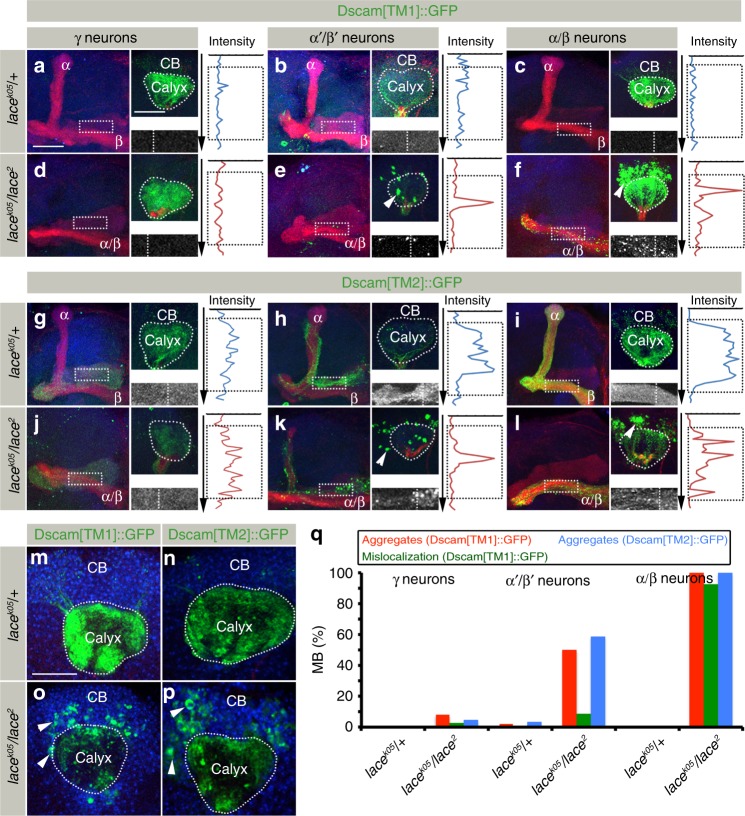
MB neuron class-specific defects of Dscam localization in *SPT* mutants. **a**–**l** Distribution of Dscam[TM1]::GFP (**a**–**f**) and Dscam[TM2]::GFP (**g**–**l**) in *lace*^*k05*^*/* *+* ((**a**–**c**) and (**g**–**i**)) and *lace*^*k05*^*/lace*^*2*^ ((**d**–**f**) and (**j**–**l**)) genetic background expressed using class-specific Gal4 expression lines, *R16A06-Gal4* (γ neurons), *R26E01-Gal4* (α′/β′ neurons), *R65G04-Gal4* (α/β neurons). While images **a**–**l** represent the axonal half of the MB, the corresponding somato-dendritic area is represented in right upper insets. The white dotted rectangle represents the Dscam distribution in the axons, zoomed in and shown in the right lower inset. White dotted line on the right lower inset represents the line along which the line intensity profile was generated from a single confocal plane. The length of the inset is represented by the dotted black rectangle on the intensity profile. γ neurons show no change in the somato-dendritic or axonal distribution of Dscam[TM1]::GFP and Dscam[TM2]::GFP (**a**, **d**, **g**, **j**, **q**). α′/β′ and α/β neurons show formation of somato-dendritic aggregates with Dscam[TM1]::GFP mislocalizing to the axonal compartments (**e**, **f**, **q**) and Dscam[TM2]::GFP showing a irregular distribution in the axons (**k**, **l**, **q**). *n*(**a**) = 32, *n*(**b**) = 48, *n*(**c**) = 40, *n*(**d**) = 38, *n*(**e**) = 46, *n*(**f**) = 34, *n*(**g**) = 24, *n*(**h**) = 32, *n*(**i**) = 34, *n*(**j**) = 22, *n*(**k**) = 34, *n*(**l**) = 38. Scale Bar: 25 μm. Green: GFP, Red: FasII, Blue: N-Cad. Arrowheads indicate somatic aggregates of Dscam. **m**–**p** Single plane confocal images of somato-dendritic area (in the genotypes indicated) co-stained with TOTO-3 antibody to label the nuclei showed that Dscam[TM1]::GFP (**o**) and Dscam[TM2]::GFP (**p**) aggregates (arrowheads) are formed in the cell body region with a corresponding decreased localization in the dendritic compartment (white dotted line) in *SPT* mutants. Green: GFP, Blue: TOTO-3. Scale Bar: 25 μm. **q** Percentage of MB showing somatic aggregates and axonal mislocalization of Dscam[TM1]::GFP and Dscam[TM2]::GFP in different MB neuronal classes

As only subtypes α′/β′ and α/β of MB are morphologically affected in *SPT* mutants, we wondered whether there is a correlation between morphological phenotypes and Dscam distribution defects. The analysis of Dscam localization in MB neuron subtypes revealed a somatic accumulation of Dscam[TM1]/[TM2] and axonal Dscam[TM1] mislocalization in α′/β′ and α/β neurons, but no effect on Dscam isoform distribution in *SPT* mutant γ neurons (Fig. 4a–l, q). The somatic Dscam protein aggregation in α′/β′ and α/β neurons is accompanied by a substantial reduction in the homogeneous dendritic and axonal membranous Dscam localization (Fig. 4a–l, line scan intensity analysis). This is most obvious following low-level transgene expression, in which all of the Dscam[TM1] protein aggregated in the somatic or axonal compartment (Fig. 4e). Nuclear staining showed no obvious difference in the overall number and position of MB neurons indicating that neuronal proliferation and survival is unaffected in *SPT* mutants (Fig. 4m–p). These data show that a reduction of sphingolipids leads to Dscam protein aggregation in the cell bodies of neurons, accompanied by a reduction of Dscam[TM1] and Dscam[TM2] at their isoform-specific dendritic and axonal localization.

### Axonal protein localization requires sphingolipids

**Fig. 5:**
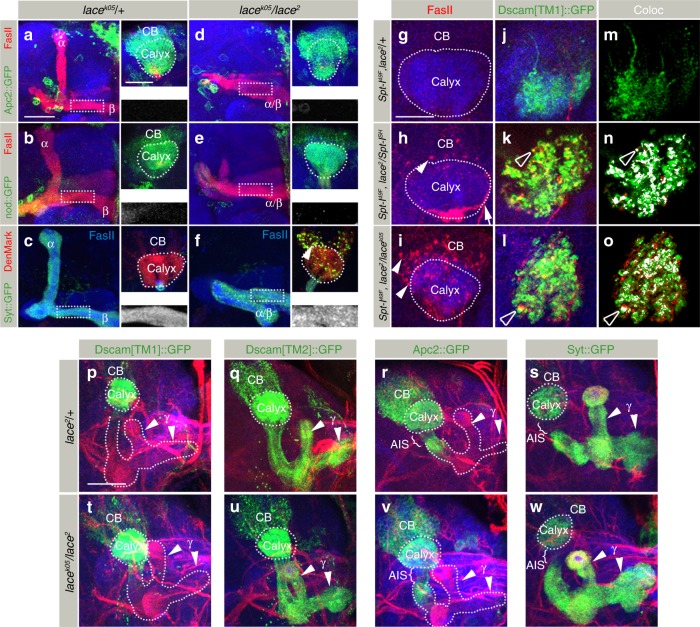
Neuronal polarity is not affected in *SPT* mutant. **a**–**f** Distribution of various dendritic (Apc2::GFP: (**a**, **d**)) (nod::GFP: (**b**, **e**)) (DenMark: (**c**, **f**)), synaptic (Syt::GFP: (**c**, **f**)) and axonal marker (FasII: (**a**–**f**)) in *lace*^*k05*^*/* *+* (**a**–**c**) and *lace*^*2*^*/lace*^*k05*^ (**d**–**f**) expressed using *OK107-Gal4* (exception: α/β specific Gal4 (R65G04-Gal4) for DenMark). While images **a**–**f** represent the axonal half of the MB, the corresponding somato-dendritic area is represented in right upper insets. The white dotted rectangle represents the indicated GFP-tagged marker distribution in the axons, zoomed in and shown in the right lower inset. While the dendritic markers do not show a change in the somato-dendritic distribution (**a**, **b**, **d**, **e**), the synaptic marker Syt::GFP shows the formation of aggregates in *lace* mutants ((**c**, **f**), arrowhead). The axonal distribution of FasII remains unchanged (**a**–**f**). *n*(**a**) = 38, *n*(**b**) = 34, *n*(**c**) = 28, *n*(**d**) = 36, *n*(**e**) = 18, *n*(**f**) = 38. **g**–**o** Somato-dendritic region of the indicated *SPT* mutant genotypes without (**g**–**l**) or with (**j**–**o**) Dscam[TM1]::GFP expression and anti-FasII staining. **m**, **n**, **o** represent processed (**j**, **k**, **l**) images for visualization of colocalization (White) of Dscam[TM1] (Green) and FasII (Red) aggregates. FasII shows the formation of aggregates in the somato-dendritic compartment in strong *SPT* mutants ((**h**, **i**) arrowheads). In addition, a smooth membranous shift of FasII into the calyx was observed (H, arrow). Co-labeling of FasII with Dscam[TM1]::GFP shows that both molecules form mostly separate aggregates in the somato-dendritic region though some colocalization was also observed ((**k**, **l**, **n**, **o**) hollow arrowheads). *n*(**g**) = 64, *n*(**h**) = 62, *n*(**i**) = 38, *n*(**j**, **m**) = 30, *n*(**k**, **n**) = 48, *n*(**l**, **o**) = 32. **p**–**w** Distribution of dendritic (Dscam[TM1]::GFP, (**p**, **t**)) (Apc2::GFP, (**r**, **v**)), axonal (Dscam[TM2]::GFP, (**q**, **u**)), and synaptic (Syt::GFP, (**s**, **w**)) markers in third instar larval MBs in *lace*^*2*^*/* *+* (**p**–**s**) and *lace*^*2*^*/lace*^*k05*^ (**t**–**w**) driven with 201Y-Gal4. No change in the distribution of the markers was observed. Further, the AIS in the larval γ neurons seemed unperturbed in *lace* mutants (**r**, **v**, **s**, **w**). *n*(**p**) = 26, *n*(**q**) = 24, *n*(**r**) = 20, *n*(**s**) = 20, *n*(**t**) = 42, *n*(**u**) = 12, *n*(**v**) = 10, *n*(**w**) = 16. **a**–**e**, **g**–**w** Green: GFP, Red: FasII, Blue: N-Cad. Scale Bar: 25 μm

To determine the importance of sphingolipids in the general organization of MB neurons, we analyzed the intra-cellular distribution of additional compartment-specific proteins. The overall dendrite organization, as indicated by microtubule polarity (nod::GFP) as well as the compartment identity shown by the restricted localization of Apc2::GFP and DenMark::Cherry^36,37^, seems unaffected in *SPT* mutant MB neurons (Fig. 5a–f). In contrast, aggregates of FasII^38^ as well as Synaptotagmin::GFP (Syt::GFP)^39^ can be detected in the somato-dendritic compartment of MB neurons, following early sphingolipid depletion (Fig. 5f, g–i). Similar to Dscam protein aggregates, the phenotypic severity of FasII aggregation directly correlates with the allelic strength (Fig. 5g–i). Interestingly, FasII and Dscam[TM1] aggregates often colocalize but also segregate into distinct protein aggregates in the somatic compartment (Fig. 5j–o).

In addition to a normal distribution of Dscam[TM1] and Dscam[TM2] in *SPT* mutant larval γ neurons, no changes can be detected in the localization of other dendritic and axonal proteins within this neuron type (Fig. 5p–w). More importantly, the γ neuron-specific axon initial segment (AIS) region, which can be visualized by the lack of Apc2::GFP and Syt::GFP localization^37,40^, develops normally in *SPT* mutants, further supporting a sphingolipid-independent mechanism of intra-cellular protein localization in γ neurons distinct from α′/β′ and α/β neurons (Fig. 5r–s, v–w).

These data show that a reduction in sphingolipids does not perturb the overall axonal and dendritic development of MB neurons, but rather interferes with the compartment-specific protein localization. For dendrite localization, Dscam[TM1] seems most sensitive to neuronal sphingolipids compared to other dendritic proteins, whereas all of the analyzed axonal proteins depend on SPT function.

### Dscam[TM1] defines axon-dendritic membrane domains

**Fig. 6:**
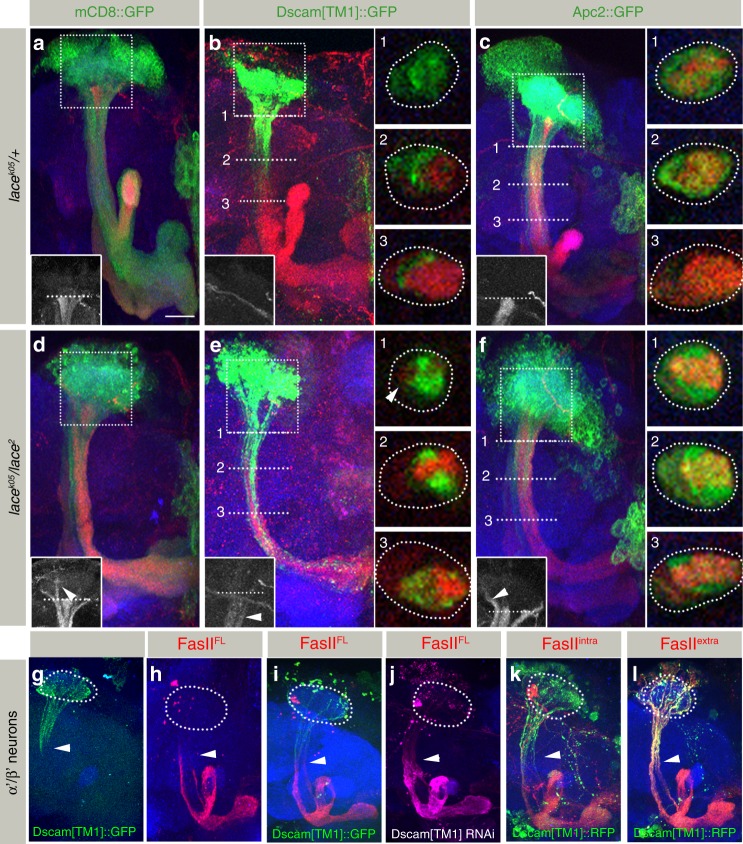
Dscam[TM1]-FasII interaction defines axonal versus dendritic domain in MB neuron. **a**–**f** MB of *lace*^*k05*^*/* *+* (**a**–**c**) and *lace*^*2*^*/lace*^*k05*^ (**d**–**f**) labeled for FasII with mCD8::GFP (**a**, **d**), Dscam[TM1]::GFP (**b**, **e**) or Apc2::GFP (**c**, **f**) expressed using *OK107-Gal4*. Inset represents FasII distribution from the area indicated by the dotted box. White dotted lines 1-3 in **b**, **c**, **e**, **f** represent three peduncular zones for analysis and the corresponding cross section of the peduncle (from a different image) is represented in the insets 1-3. In *SPT* mutants, membranous FasII shifts upwards into the dendritic compartment (inset (**d**), arrowhead). The expression of Dscam[TM1]::GFP causes axo-dendritic boundary of FasII to shift deeper into the peduncle, while retaining the exclusion from Dscam[TM1]::GFP (**b**). FasII partially regains its axo-dendritic boundary in *SPT* mutants owing to absence of membranous Dscam[TM1] ((**e**), arrowhead insets). Expression of the cytosolic dendritic marker Apc2::GFP causes no shift in FasII localization (**c**, **f**). **g**–**l** MB in the wild-type background with α′/β′ neuron-specific expression (R26E01-Gal4) of Dscam[TM1]::GFP (**g**), FasII^PEST−^ (**h**), Dscam[TM1]::GFP + FasII^PEST-^ (**i**), Dscam[TM1]::RNAi + FasII^PEST−^ (**j**), Dscam[TM1]::RFP + intra-FasII-YFP (**k**), and Dscam[TM1]::RFP + extra-FasII-YFP (**l**). Full length (FL) FasII and Dscam[TM1] maintains exclusion in α′/β′ neurons (**g**–**i**). Interestingly, Dscam[TM1] knockdown causes FasII to shift into the somato-dendritic compartment (**j**). On the other hand, intra-FasII-YFP (**k**) but not extra-FasII-YFP (**l**) shows membranous exclusion from Dscam[TM1], indicating that the membranous exclusion is mediated by interactions in the intra-cellular domain. The white arrowhead marks the axo-dendritic boundary, the white dotted circle marks calyx. *n*(**g**) = 28, *n*(**h**) = 40, *n*(**i**) = 24, *n*(**j**) = 36, *n*(**k**) = 28, *n*(**l**) = 28. **a**–**l** Green: GFP, Red: FasII, Blue: N-Cad, Scale Bar: 25 μm

Interestingly in addition to somatic aggregates, we also observed changes in the membranous distribution of axonal FasII dependent on dendritic Dscam[TM1] levels. In wild type MB neurons, FasciclinII localizes exclusively in the axonal compartment (Fig. 6a, *n* = 18). In addition to the FasII protein aggregation in the soma of *SPT* mutant MB neurons described above, a reduction in sphingolipids also leads to a shift of the FasII-positive axonal domain into the dendritic calycal region (Figs. 5h, 6d, 11/15 = 73% of MB shows calycal shift of FasII). We hypothesized that the dendritic reduction of Dscam[TM1] due to somatic aggregation in *SPT* mutants may allow axonal FasII to extend into the calyx. To test whether dendritic Dscam isoforms directly influence axonal FasII distribution, we modified Dscam[TM1] expression in wild type MB neurons. Elevated levels of Dscam[TM1] in developing MB neurons led to an extension of the Dscam[TM1] from the calycal region into the peduncle (Fig. 6b, 12/12 = 100% of MB shows Dscam[TM1] localization to section 3). Strikingly, the expansion of Dscam[TM1] into the distal peduncle results in a corresponding shift of FasII membrane localization, thereby retaining the strict exclusion of Dscam[TM1] and FasII membrane domains (Fig. 6b, 0/12 = 0% MB shows FasII between calyx and Line1). As overexpression of the cytoplasmic dendritic protein Apc2 did not affect FasII localization (Fig. 6c, f, in (f) 14/15 = 93% MB show shift of FasII to the clayx in *lace* mutants, *n*(c) = 16, *n*(f) = 15), the mutual exclusion of FasII and Dscam[TM1] might result from direct interactions within the membrane. This is supported by *SPT* mutants in which a reduced membrane integration of Dscam[TM1] allows FasII to regain its axo-dendritic boundary (Fig. 6e, inset 1 arrowhead, 6/11 = 54.5% MB FasII regains localization between calyx and section 1 as compared to 0% in B). To test whether the mutual exclusion of Dscam[TM1] and FasII is a result of the interaction at the extra- or intra-cellular domains, we expressed fluorescent-tagged truncated FasII constructs in α′/β′ neurons^41^. Full length FasII and Dscam[TM1] maintains mutual exclusion of the two protein domains within the neuronal membrane (Fig. 6g–i). Additionally, RNAi-mediated Dscam[TM1] knockdown causes a shift of FasII localization into the somato-dendritic compartment, further confirming the instructive role of Dscam[TM1] in maintaining the axo-dendritic boundary (Fig. 6j). Interestingly, upon truncation of the intra-cellular domain of FasII its membranous exclusion by Dscam[TM1] and axon-specific localization is lost (Fig. 6k, l). These results support an instructive function of Dscam[TM1] in defining the somato-dendritic compartment by excluding axonal membrane molecules via intra-cellular interactions.

### Dscam[TM1] interferes with Dscam[TM2] function in *SPT* mutant

**Fig. 7:**
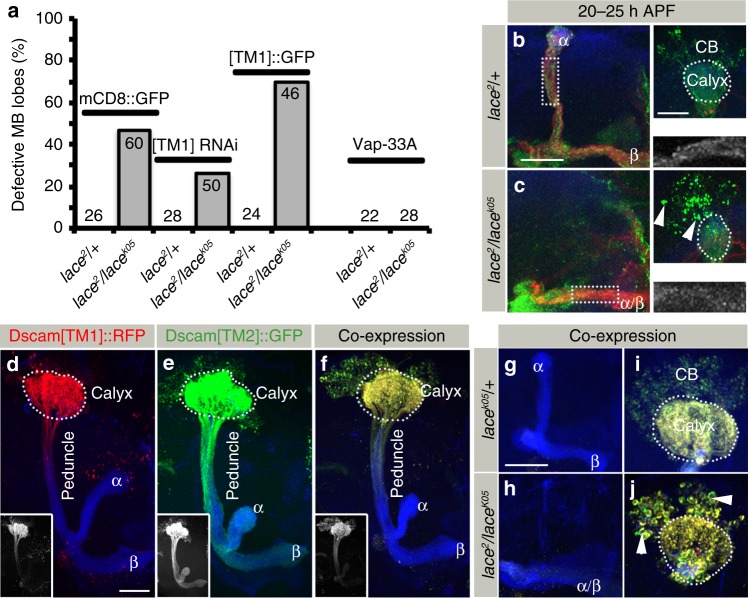
Dscam[TM1] interferes with Dscam[TM2] function in *SPT* mutant MB neurons. **a** Percentage of defective MB lobes in different indicated genetic backgrounds. In *SPT* mutants, knocking-down of Dscam[TM1] and expressing Vap33 using Dscam-Gal4 reduces axonal targeting defects while overexpression of Dscam[TM1] enhances it. Numbers on the bars represent number of MB analyzed. **b**, **c** Distribution of Dscam[TM2]::GFP at 20–25 h APF in the MB of *lace*^*2*^*/* *+* (**b**) and *lace*^*2*^*/lace*^*k05*^ (**c**) expressed using 201Y-Gal4. While images **b**, **c** represent the axonal half of the MB, the corresponding somato-dendritic area is represented in right upper insets. The white dotted rectangle represents the Dscam[TM2]::GFP distribution in the axons, zoomed in and shown in the right lower inset. Green: Dscam[TM2]::GFP, Red: FasII, Blue: N-Cad. Arrowheads indicate somatic aggregates of Dscam. *n*(**b**) = 14, *n*(**c**) = 12. **d**–**f** MB expressing Dscam[TM1]::RFP (**d**), Dscam[TM2]::GFP (**e**) and Dscam[TM1]::RFP + Dscam[TM2]::GFP (**f**) using a α/β neuron-specific Gal4 (R65G04-Gal4). Insets show single channel fluorescence of Dscam[TM1] (**d**) /Dscam[TM2] (**e**) /Dscam [TM2] (**f**). *n*(**d**) = 26, *n*(**e**) = 26, *n*(**f**) = 28. **g**–**j** On expressing Dscam[TM1]::RFP (Red) with Dscam[TM2]::GFP (green) in *lace*^*k05*^*/* *+* (**g**, **i**) and *lace*^*2*^*/lace*^*k05*^ (**h**, **j**) background with α/β specific Gal4 (R65G04-Gal4), Dscam[TM2] fails to localize to axons. *n*(**g**, **i**) = 28, *n*(**h**, **j**) = 30. **d**–**j** Green: Dscam[TM2]::GFP, Red: Dscam[TM1]::RFP, Blue: FasII, Arrowheads indicate somatic aggregates. Scale Bar: 25 μm

As Dscam[TM2] is required for the segregation of axon branches in developing MB neurons^20–23^ and Dscam[TM1] becomes mislocalized to the axonal compartment in *SPT* mutants, we wondered whether Dscam[TM1] might interfere with Dscam[TM2] mediated axon segregation in a sphingolipid depleted background. Therefore, we analyzed the effect of changing the levels of Dscam[TM1] on the penetrance of axon lost-lobe phenotype in *SPT* mutants. A reduction in levels by targeted RNAi as well as enhanced expression of Dscam[TM1] does not affect axonal branch segregation of wild-type MB neurons (Fig. 7a). In contrast, Dscam[TM1] knockdown in *SPT* mutants leads to a substantial rescue of the “lost-lobe” MB phenotype whereas elevated Dscam[TM1] expression enhances the *SPT* mutant MB lobe defects (Fig. 7a). These results suggest that, following a reduction in sphingolipids, Dscam[TM1] directly influence Dscam[TM2] activity in MB neurons.

Next we tested if the perturbed Dscam[TM2] function is a result of reduced axonal localization. In *SPT* mutants a significant reduction in the axonal Dscam[TM2] levels can be detected in newly born α/β axons, which correlates with the axon branch defect (Fig. 7b, c). Furthermore, overexpression of Vap33, a transport protein recently identified to mediate Dscam[TM2] axon localization^42^, rescues the axonal branching defects of *SPT* mutants (Fig. 7a). These data support a mechanism in which, following sphingolipid reduction, Dscam[TM1] interferes with the axonal localization of Dscam[TM2] in growing MB neurons.

To analyze the interaction of Dscam[TM1] and Dscam[TM2] more directly, we expressed differentially tagged isoforms and studied their distribution in developing MB neurons. In wild-type neurons, the singular expression of Dscam[TM1] and Dscam[TM2] leads to the compartment-specific distribution into MB dendrites and axons, respectively (Fig. 7d, e). In contrast, the co-expression of Dscam[TM1] and Dscam[TM2] result in a complete redistribution of Dscam[TM2] from the axonal into the dendritic compartment (Fig. 7f–j) showing that upon isoform interaction, Dscam[TM1] prevents Dscam[TM2] translocation into axons. This suggests a critical role for sphingolipids in regulating intra-cellular Dscam isoform sorting.

### Dscam aggregates in *SPT* mutants escape protein degradation

**Fig. 8:**
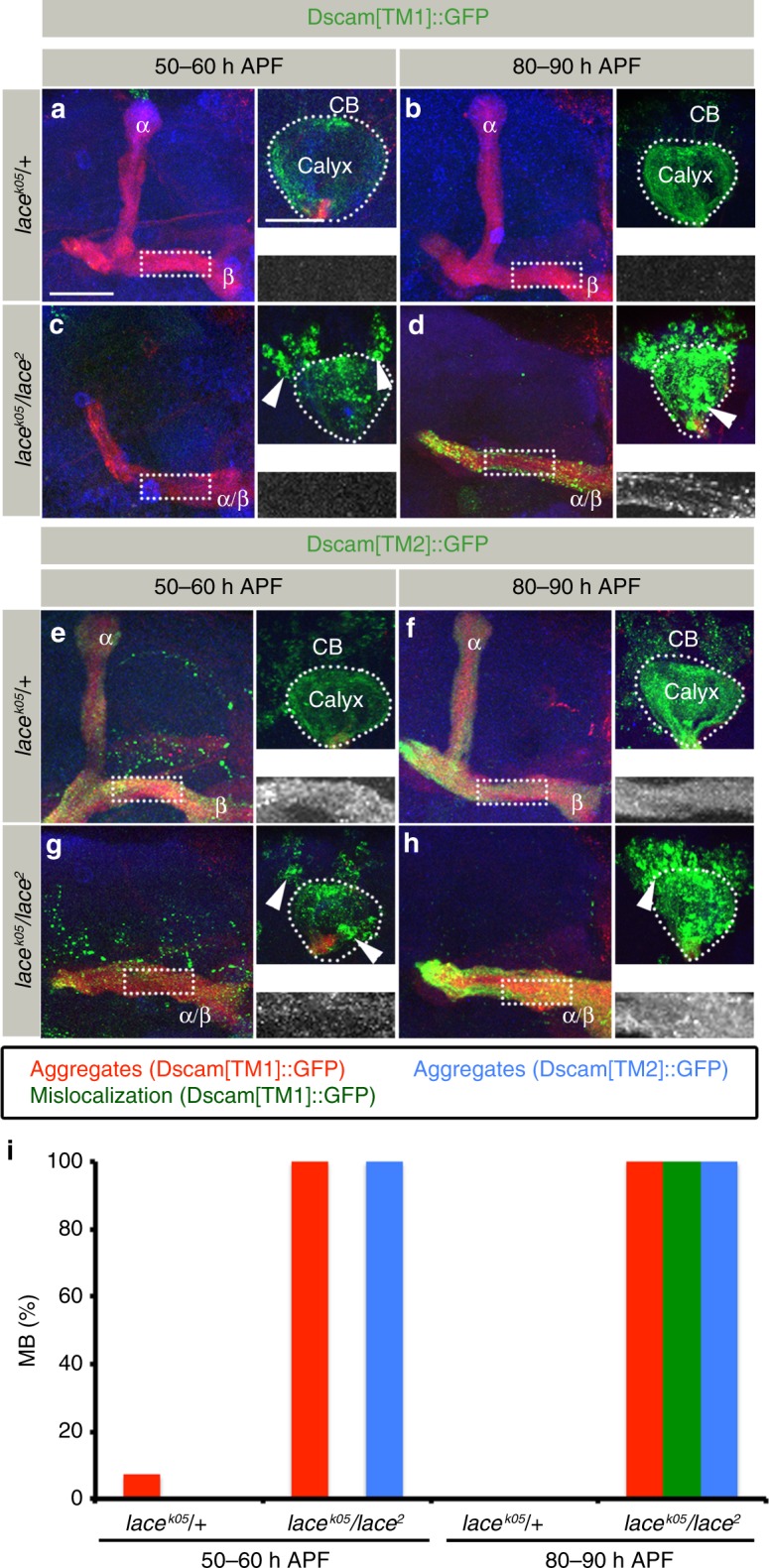
Dscam aggregates show intra-cellular translocation in *SPT* mutants. **a**–**d** Distribution of Dscam[TM1]::GFP in *lace*^*k05*^*/* *+* (**a**, **b**) and *lace*^*2*^*/lace*^*k05*^ (**c**, **d**) at two pupal developmental stages: 50–60 h APF (**a**, **c**) and 80–90 h APF (**b**, **d**) expressed using α/β neuron-specific Gal4 (*R65G04-Gal4*). While images **a**–**d** represent the axonal half of the MB, the corresponding somato-dendritic area is represented in right upper insets. The white dotted rectangle represents Dscam[TM1]::GFP distribution in the axons, zoomed in and shown in the right lower inset. Dscam[TM1]::GFP shows an early formation of aggregates ((**c**), arrowheads) followed by axonal mislocalization (**d**) in *lace* mutants. *n*(**a**) = 14, *n*(**b**) = 14, *n*(**c**) = 14, *n*(**d**) = 12. **e**–**h** Distribution of Dscam[TM2]::GFP in *lace*^*k05*^*/* *+* (**e**, **f**) and *lace*^*2*^*/lace*^*k05*^ (**g**, **h**) at two pupal developmental stages: 50–60 h APF (**e**, **g**) and 80–90 h APF (**f**, **h**) expressed using α/β neuron-specific Gal4 (R65G04-Gal4). Dscam[TM2]::GFP shows reduced axonal localization early in development with formation of somato-dendritic aggregates (Compare (**e**) and (**g**), arrowheads) in *SPT* mutants. This is followed by a non-homogeneous distribution in the axons (**h**). *n*(**e**) = 18, *n*(**f**) = 8, *n*(**g**) = 8, *n*(**h**) = 20. **i** Percentage of MB showing somatic aggregates and axonal mislocalization of Dscam[TM1]::GFP and Dscam[TM2]::GFP in MB at different developmental times. **a**–**h** Green: GFP, Red: FasII, Blue: N-Cad. Arrowheads indicate somatic aggregates of Dscam. Scale Bar: 25 μm

To get further insights into the mechanism of sphingolipid-sensitive Dscam[TM1]-Dscam[TM2] interactions, we followed the Dscam isoform distribution during MB development. The expression of Dscam[TM1] in developing wild-type α/β neurons (50 h APF) leads to a homogeneous protein distribution in the dendritic membrane and no localization in axons can be detected (Fig. 8a). In contrast, in *SPT* mutant neurons, Dscam[TM1] largely fails to localize to the dendritic compartment and Dscam[TM1] protein aggregates become visible in the cell bodies of MB neurons (Fig. 8c, i). Subsequently, Dscam[TM1] aggregates translocate into axons and accumulate in the distal peduncle lobe regions (compare Fig. 8b, d). Similar somatic Dscam[TM1] aggregate formation and axonal mislocalization could be observed in more mature MB neurons of *SPT* mutants. Here, pulses of Dscam[TM1] expression in the adult brain using the TARGET system (Gal80^ts^)^43^, led to the protein aggregation phenotype, indicating that sphingolipid-dependency is not restricted to early neuronal differentiation, but may also affect functional circuits (Supplementary Figure 6).

In contrast to the sequential appearance of Dscam[TM1] aggregates in the soma and axons of *SPT* mutant neurons, Dscam[TM2] aggregates are visible in axons already at an early stage of neuronal development, with an overall reduction of axonal Dscam[TM2] (Fig. 8e, g, i). In later stages of development, Dscam[TM2] aggregates are highly enriched in the somatic and axonal compartment (Fig. 8f, h, i).

**Fig. 9:**
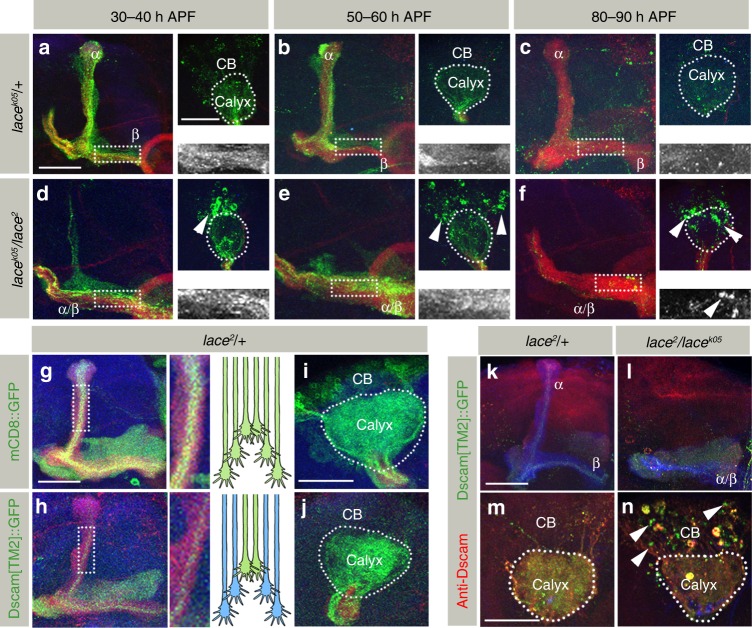
Dscam aggregates in *SPT* mutants escape protein degradation. **a**–**f** Distribution of Dscam[TM2]::GFP in *lace*^*k05*^*/* *+* (**a**–**c**) and *lace*^*2*^*/lace*^*k05*^ (**d**–**f**) at different stages of pupal development, 30–40 h (**a**, **d**), 50–60 h (**b**, **e**), and 80–90 h (**c**, **f**) expressed using Rubin Gal4 (*R30F11-Gal4)*. While images **a**–**f** represent the axonal half of the MB, the corresponding somato-dendritic area is represented in right upper insets. The white dotted rectangle represents Dscam[TM2]::GFP distribution in the axons, zoomed in and shown in the right lower inset. In *lace*^*k05*^*/* *+* expression of Dscam[TM2] using *R30F11-Gal4* starts to decline by 50–60 h APF (**e**) and is nearly not detectable at 80–90 h APF (**f**). On the other hand, *lace*^*k05*^*/lace*^*2*^ shows formation of aggregates by 30–40 h APF ((**d**), arrowhead), which grow by 50–60 h APF ((**e**), arrowheads) and are stable later in the development (80–90 h APF, (**f**), arrowhead), although membranous Dscam[TM2] is degraded. *n*(**a**) = 14, *n*(**b**) = 22, *n*(**c**) = 12, *n*(**d**) = 8, *n*(**e**) = 8, *n*(**f**) = 10. Green: Dscam[TM2]::GFP, Red: FasII, Blue: N-Cad. Arrowheads indicate somatic aggregates of Dscam[TM2]::GFP. **g**–**j** The relatively fast turn-over dynamics of Dscam[TM2] is also evident when comparing the axonal distribution of mCD8::GFP (**g**, **i**) with Dscam[TM2]::GFP (**h**, **j**). Dscam[TM2]::GFP shows much restricted distribution in terms of expression in newly developed neurons (201Y-Gal4) when compared to membranous marker mCD8::GFP. The white dotted rectangle represents the axonal area zoomed in the inset. Green: mCD8::GFP, Red: FasII, Blue: N-Cad. **k**–**n** The Dscam[TM2] aggregates are only partially recognized by the Dscam antibody ((**n**), arrowheads), in contrast to the membranously localized Dscam[TM2]::GFP, which is fully accessible to the Dscam antibody in *lace*^*2*^*/* *+* (**k, m**) and *lace*^*k05*^*/lace*^*2*^ (**l**). *n*(**k**, **m**) = 28, *n*(**l**, **n**) = 30. Green: Dscam[TM2]::GFP, Red: anti-Dscam, Blue: N-Cad. Scale Bar: 25 μm

Using a transient low-level expression of Dscam[TM2], the temporal dynamics of Dscam protein aggregates were visualized in more detail (Fig. 9). Following the onset of Dscam[TM2] expression in wild-type neurons, a homogeneous Dscam[TM2] distribution could be detected in the axonal compartment, with significantly lower Dscam[TM2] levels within the dendrites and no protein localization in the neuronal somata (Fig. 9a, b). The membrane-localized Dscam[TM2] rapidly disappeared when transgene expression stopped, indicating a fast Dscam protein turn-over (Fig. 9c). In addition, the direct comparison of protein perdurance between Dscam[TM2] and mCD8 in newly extending α/β neurons using the transient *201Y-Gal4* driver line revealed a rapid degradation of Dscam[TM2] following axon extension (Fig. 9g–j). In *SPT* mutants, Dscam[TM2] shows a fast aggregation in neuronal cell bodies and these protein aggregates remain stable independently of transgene expression (Fig. 9d–f). Interestingly, membrane-localized protein in *SPT* mutants was removed in a similar temporal pattern as in wild-type neurons, indicating that protein dynamics strongly depend on the intra-cellular localization (Fig. 9d–f). Furthermore, in contrast to membrane-integrated Dscam[TM2], most aggregates of Dscam[TM2] are not recognized by an anti-Dscam antibody against the intra-cellular domain^16^, possibly due to substantial conformational changes (Fig. 9k–n). These data show that Dscam protein is characterized by a rather short half-life upon membrane integration and its degradation is impaired in sphingolipid-deprived neurons due to formation of cytoplasmic aggregates.

### Human *SPT*^HSAN-1^ mutations lead to Dscam aggregation

**Fig. 10:**
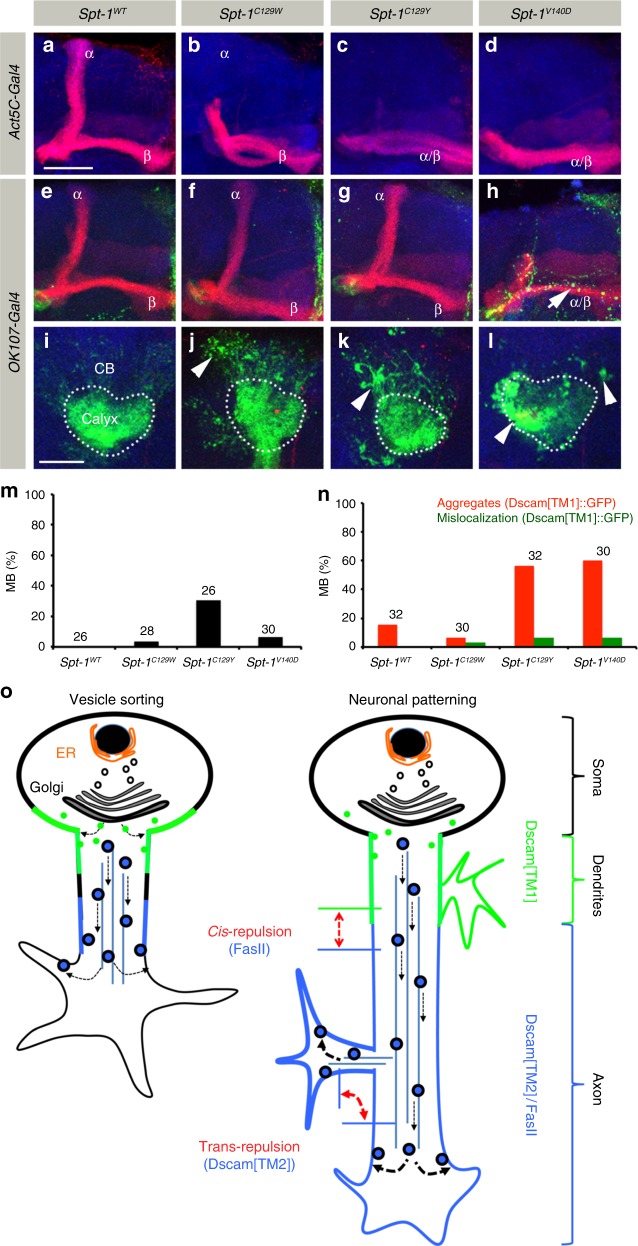
Human *SPT*^*HSAN-1*^ mutations lead to Dscam aggregation and mislocalization. **a**–**d** MB axonal morphology in *Drosophila* ubiquitously expressing (Act5c-Gal4) *Spt-I*^*WT*^ (**a**) or the mutations *Spt-I*^*C129W*^(**b**), *Spt-I*^*C129Y*^ (**c**), and *Spt-I*^*V140D*^ (**d**) homologous to human HSAN-1 mutations. MB show axonal morphology defects in HSAN-1 associated mutations (**b**–**d**). **e**–**l** MB-specific overexpression (*OK107-Gal4*) of *Spt-I* mutant constructs with Dscam[TM1]::GFP shows formation of aggregates (arrowheads (**j**–**l**)) and also axonal mislocalization (**h**, arrow). Green: Dscam[TM1]::GFP, Red: FasII, Blue: N-Cad. Scale Bar: 25 μm. **m** Percentage of MB showing axonal morphology defects in the background of expression of various human *SPT* mutations. **n** Percentage of MB showing aggregation and mislocalization of Dscam[TM1]::GFP in the background of expression of various human *SPT* mutations. Numbers on the bars represent number of MB analyzed. **o** Model for the role of sphingolipids in the segregation of Dendritic vs. axonal proteins. Sphingolipids regulate segregation of Dendritic (Dscam[TM1] (green)) and axonal (Dscam[TM2]/FasII (blue)) proteins by separating them into vesicles with low (no boundary) or high level (black boundary) of sphingolipids, respectively. These vesicles are then specifically targeted to either dendrites or axons. *Cis*-membrane interactions between Dscam[TM1] and axonal proteins can define the axo-dendritic boundary during the development. On the other hand, transmembrane homophilic repulsion between Dscam[TM2] isoforms is important for the proper development of axonal morphology

Recently, mutations in the human SPT subunits SPTLC1/SPTLC2 have been linked to HSAN-1 (Hereditary Sensory and Autonomic Neuropathy type-1)^24,25,44,45^. To determine if these mutations affect neuronal development, we expressed the corresponding *SPT* mutant forms of the *Drosophila* protein in developing MBs (Fig. 10). Two independent point mutations of a conserved cysteine [C133W (C129W in *Drosophila*) and C133Y (C129Y in *Drosophila*)] and one in conserved valine [V144D (V140D in *Drosophila*)] of SPTLC1 are associated with severe forms of late-onset neurodegeneration^25,45^. Interestingly, *Spt-I*^*B2*^, one of the mutations isolated in our genetic mosaic screen, affects a neighboring conserved glycine (G127E) (Fig. 1a). Following the global expression of *Spt-I*^*C129W*^, *Spt-I*^*C129Y*^ and *Spt-I*^*V140D*^ throughout development, axon branch segregation defects of MB neurons could be observed (Fig. 10b-d, m). In contrast, *Spt-I*^*WT*^ expression did not influence neuronal development (Fig. 10a, m). In addition, MB-specific expression of *Spt-I*^*C129W/Y*^ and *Spt-I*^*V140D*^ result in impaired Dscam[TM1], as well as FasII transport and the formation of corresponding protein aggregates (Fig. 10e–l, n). These data point to aggregation of neuronal cell adhesion molecules as a common subcellular neuronal defect in developmental and disease-linked mutations, providing novel mechanistic insights into the process of HSAN-1 related neurodegeneration.

## Discussion

Neurons provide an excellent example for the coupling of morphological and functional polarity, maintained by a strict control of polarized protein distribution via vesicle transport^1–3^. A major structural component of transport vesicles in addition to proteins are lipids. Here we show that sphingolipids are critical to organize early protein targeting to axonal and dendritic processes in neurons. Mutations in *Drosophila SPT*, a key enzyme in de novo sphingolipid biosynthesis^26^, lead to a severe reduction in membrane localization of dendrite and axon-specific Dscam isoforms. The impairment of axonal Dscam[TM2] localization disrupts axon branch segregation in extending MB neurons^15^. On the other hand, localized Dscam[TM1] membrane integration, which seems to be dispensable for dendrite patterning in MB^15^, is critical for defining the initial axo-dendritic boundary within neuronal membranes. As sphingolipids are enriched in the trans-golgi network and plasma membrane^6^, we propose a model for the role of sphingolipids in localization of dendritic vs. axonal proteins during neuronal development (Fig. 10o). In extending neurons, Dscam[TM1] and Dscam[TM2] are sorted depending on their localization in sphingolipid low or rich membrane vesicles formed at the trans-golgi network and afterwards targeted to the proximal or distal axonal membrane compartments, respectively. In addition to the development of proper neuronal morphology depending on homophilic repulsion between similar Dscam[TM2] isoforms^12^ at the tip of extending axons, Dscam[TM1]-mediated *cis*-interactions within the proximal axon segment restrict axonal protein localization thereby defining the initial axo-dendritic boundary. This model is strongly supported by our observation that Dscam[TM2] is enriched in sphingolipid-rich detergent resistant membrane (DRM) fraction from adult *Drosophila* brain (Supplementary Figure 7).

Consistent with the previous reports, we detected Cer(d14:1/20:0) and Cer(d14:1/22:0) as the major ceramide species in *Drosophila* among other ceramide species with different Fatty acyl chain lengths and saturations^29,46,47^. *SPT* mutants in *Drosophila* have been previously shown to have reduced levels of sphingolipids^29–33^. Using mass-spectrometric analysis, we found a significant reduction in the levels of total ceramide, resulting from reduction in amount of 5 ceramide species. Certain ceramide subtypes have been implicated in influencing different biological activities e.g., ceramide (d18:1/16:0) regulates release of CytC from mitochondria whereas ceramide (d18:1/18:0) potentiates mitophagy^48^. In *SPT* mutants, we could not detect a direct correlation between the affected ceramide species and the axonal phenotype, suggesting that the overall reduction in the levels of ceramides results in disruption of axon-dendritic protein sorting. On the other hand, we did not find any reduction in the total levels of membrane phospholipid PC and another sphingolipid Ceramide-phosphoethanolamine (Cer-PE), a downstream product of ceramide. This suggests that for Cer-PE, some alternative pathways or feedback mechanisms can compensate for the reduced amounts of input ceramide^49^.

During neuronal development, polarized transport is established even before axon or dendrite specific microtubule polarity becomes visible^5^. Here we show that sphingolipids play an important role in initial axo-dendritic sorting of Dscam isoforms required for initial patterning of growing neurons. Interestingly, dynein–dynactin-based retrograde transport has been shown to influence Dscam isoform localization but, in contrast to the sphingolipid-mediated mechanisms described here, controls the maintenance of polarized protein following initial sorting and does not influence neuronal pattering^50^. During dendritic development, the primary function of Dscam is self neurite repulsion^13^ whereas in axonal development, Dscam regulates segregation, targeting, collateral formation, as well as arbor size^14–19^. Not only the function is compartment specific, it also depends on the expression levels of Dscam^18,19^. Our observations suggest that, in the absence of sphingolipids, membrane integration of Dscam transmembrane isoforms is severely reduced. Dscam[TM2] dependent axonal morphology defects in *SPT* mutants are directly influenced by changes in the levels of dendritic Dscam[TM1] indicating inappropriate isoform interactions in the absence of sphingolipids. Therefore initial sphingolipid-dependent sorting of Dscam[TM1] and Dscam[TM2] is critical to allow axonal targeting of Dscam[TM2] and for subsequent development of axonal morphologies.

Various observations suggest that axonal transport depends on sphingolipid-rich vesicles in contrast to vesicular transport into dendrites. By using chemical inhibitors it has been shown that axonal growth depends on sphingolipid biosynthesis and axonal proteins fractionate in detergent insoluble sphingolipid-rich fractions^10,11^. Furthermore, ER to Golgi transport with vesicles of low sphingolipid content maintains membrane transport to dendrites and is important for dendritic but not axonal morphology^51^. Interestingly, the increased width of sphingolipid/cholesterol-rich membranes prefer the integration of proteins with longer transmembrane domains^7^. The transmembrane/juxta-membrane domain of Dscam[TM2] is substantially larger than the one of Dscam[TM1] (71 versus 56 a.a.), which could favor its segregation into sphingolipid-rich vesicles in MB neurons. Supporting the role of sphingolipids in axonal transport, the localization of all tested axonal proteins were affected in *SPT* mutants while dendritic markers, except Dscam[TM1], showed no change in compartment-specific distribution (Fig. 5). Thus, similar to apical transport in epithelial cells, axonal transport in neurons depend on sphingolipids while sphingolipid low vesicles target to dendritic or baso-lateral membranes in neurons or epithelial cells, respectively^52^.

One of the surprising findings of our study is the neuron-type specific requirement of sphingolipids in compartment-specific protein sorting. In *SPT* mutants, γ neurons showed no developmental morphological defects and reduced Dscam isoform distribution as opposed to α′/β′ or α/β neurons (Figs. 2, 4, 5). Interestingly in *Dscam* mutants γ neurons also display a wild-type morphology whereas development of α′/β′ or α/β neurons is severely affected^15,22^. Although all MB neurons are deriving from a single neuroblast, developmentally and molecularly γ neurons are substantially different from the later-born α′/β′ and α/β neurons. For example, γ neurons undergo developmental pruning and regrowth dependent on dynamic regulation of FasII, not shown by α′/β′ or α/β neurons^53,54^. A neuronal compartment similar to the AIS in vertebrates has been reported specifically for γ neurons in MB of *Drosophila*^40^, which is not affected by the absence of sphingolipids. These data suggest the existence of different protein sorting mechanisms even between neurons derived from the same precursors and might support neuron-type specific function in MB-associated learning and memory formation^55^.

In bi- and multipolar neurons, a neurite is selected during early development to become the axonal compartment and subsequently the axon-somatic boundary is defined by AIS assembly^1,56^. The situation is different in uni-polar neurons where the axo-dendritic boundary has to be specified within a single neurite^2^. Developmental studies have shown that MB neurons as well as projection neurons in the olfactory system first extend an unbranched axon towards their target region before co-lateral dendritic processes are initiated at the proximal region, raising the question about the underlying mechanism of initial protein targeting for dendrite growth and patterning^57,58^. Our results described here suggest a 2-step process in which first, Dscam[TM1] vesicles of low sphingolipid content escape anterograde axonal transport and integrate into the proximal neuronal cell membrane (Fig. 10o) and second, membrane-integrated Dscam[TM1] directly determine the proximal extension of axonal proteins via mutual exclusion (Fig. 10o). Although we could not test directly a putative complementary instructive function of Dscam[TM2] due to a gain-of-function effect, we speculate that FasII is a readout for the axonal compartment defined by the interactions of Dscam[TM2] with Dscam[TM1]. *cis-*repulsion between identical Dscam isoforms targeted to dendritic [TM1] or axonal [TM2] compartments can define the axo-dendritic boundary, possibly via activation of differential signaling downstream of the two trans/juxta-membrane Dscam isoforms^14,59^.

Within growth cones of extending neurons rapid protein degradation balances protein synthesis to ensure context-dependent responsiveness to external guidance cues^60^. Upon targeted membrane localization in growing neurons, Dscam undergoes fast protein turn-over as revealed by growing axons (MB core) specific staining of Dscam antibody^22^. The proposed function of Dscam isoform hyper-variability coupled with isoform-specific interactions allow cell-intrinsic neurite repulsion while ignoring neurites from neighboring neurons^12^. Self versus non-self recognition could be supported by down-regulating Dscam protein levels immediately after a neuron has acquired its final morphology. The presence of a strong PEST motif (PEST score: 14.72) in Dscam suggests ubiquitin-proteasome mediated removal^61^. Interestingly, reduction of sphingolipids result in Dscam molecules escaping fast intrinsic degradation, leading to formation of protein aggregates, which translocate from somato axons, It has been shown before that depending on the context, sphingolipids can nucleate or prevent protein aggregation^62^. These experiments suggest that sphingolipid-dependent segregation of Dscam isoforms is essential to prevent formation of aggregates. Further experiments are needed to address whether the Dscam aggregates are isoform specific or are formed as a result of interaction between the different isoforms.

Human genetic disorders associated with lipid metabolism is an increasing group of inherited form of diseases^63^. HSAN-1, caused by dominant mutations in SPT, is manifested as peripheral sensory loss and axonal degeneration^24,25,45^. The current understanding of HSAN-1 pathology involves formation of toxic metabolic intermediates due to changed substrate specificity of SPT^27,64,65^. We found that the expression of *SPT* mutations associated with severe forms of human HSAN-1 pathology, result in neuronal patterning defects and the formation of intra-cellular aggregates of cell surface molecules.

Formation of endogenous protein aggregates is associated with a number of neurodegenerative disorders^66^. In Alzheimer’s disease, sphingolipid-rich microdomains can act as a nucleation sites for amyloid β formation, whereas the effect is opposite in case of Prion proteins^62,66^. On the contrary, changes in the levels of various sphingolipid sub types has been observed in Alzheimer’s disease^62,66^. Thus, sphingolipids being in close proximity with membranous amyloidogenic proteins, influence their biology and thus progression of neurodegenerative disorders. Until now, the formation of endogenous protein aggregates has not been associated with the pathology of inherited disorders of sphingolipid metabolism^63^. We hypothesize that the stable Dscam aggregates formed in *SPT* mutants/HSAN-1 might interfere with synaptic activity and eventually result in axonal degeneration associated with HSAN-1. It has recently been shown that the expression of HSAN-1 associated allele *Spt-I*^*C129W*^ in peripheral neurons of *Drosophila* larvae leads to reduced nociception^33^. On the other hand, a glia specific knockdown of SPT subunit Lace causes defective glial ensheathment of peripheral nerves in *Drosophila*^30^. Thus sphingolipids may function not only in neuronal development but also in interaction of neuron and glia, important for neuronal maintenance and function. Finally, our findings regarding the neuron-type specific dependence on sphingolipids for protein sorting could provide novel insights into the differential vulnerability in the context of neurodegeneration.

## Methods

### *Drosophila* strains

The flies were reared at 25 °C in vials containing standard cornmeal agar medium. *Drosophila melanogaster* strains were generally obtained from Bloomington *Drosophila* Stock Center (BDSC), as well as Vienna *Drosophila* Resource Center (VDRC). Detailed information about the transgenic flies and the exact genotypes of flies used in different experiments is provided as Supplementary methods and Supplementary Table 1 in the Supplementary information file.

### Clonal analysis

Single cell clones were generated using different genetic approaches. Mosaic analysis with repressible cell marker (MARCM)^67^ was used to generate single homozygous mutant clones in a heterozygous mutant background by mitotic recombination. Flybow (*FB1.1B*)^68^ or a UAS > CD2 > CD8::GFP Flp-out cassette^69^ was used to visualized single cells in wild type, as well as mutant background. To generate single cell clones with flybow or Flp-out cassette, mid-pupae were heat shocked for 20 min or 30 sec at 38 °C, respectively. The pupae were then allowed to further develop at 25 °C and were dissected after eclosion.

### Temporal expression of Dscam[TM1] using the TARGET system

Flies of the desired genotypes were reared at 18 °C and then shifted to 29 °C for a 48 h period within 1–2 days of eclosion. After 48 h, the flies were dissected and the brains processed for immunohistochemistry.

### Sphingosine supplementation of the food

Mutant larvae were reared on standard cornmeal agar medium, without (−) or with ( + ) D-sphingosine (10 μM, sigma, cat. No.- S7049) supplementation. After eclosion, the flies were dissected within 2 days and the brains processed.

### Immunohistochemistry

*Drosophila melanogaster* male and female flies were used 3–5 days post eclosion, unless otherwise specified. Brains and wing discs were dissected in phosphate-buffered saline (PBS) and fixed in 2% paraformaldehyde (PFA) in PBS for 60 min. Samples were rinsed once and washed (4 × 15 min) with PBS-T (PBS containing 0.3% Triton X-100) with constant shaking on a horizontal shaker. The samples were then blocked for 1 h (10% Goat serum in PBS-T) before adding the primary antibody and incubating over-night at 4 °C. This was followed by washing with PBS-T (4 × 15 min) and incubating with secondary antibody over-night at 4 °C. After washes (PBS-T, 4 × 15 min), the brains were mounted in Vectashield® antifade mounting medium (Vector laboratories). As primary antibodies rat anti-DN-cadherin (DN-Ex #8, DSHB, T. Uemura, 1:20), mouse anti-Fas II (1D4, DSHB, C. Goodman, 1:5), mouse anti-Flamingo (Flamingo#74, DSHB, T. Uemura, 1:20), mouse anti-CD2 (AbD Serotec® MCA154G, 1:1000), and rabbit anti-Hemagglutinin (HA, Sigma-Aldrich, 1:100) antibody were used. Rabbit anti-Dscam antibody^16^ (1:1000) was kindly provided by Prof. Dietmar Schmucker. Secondary antibodies (Alexa Fluor®, Molecular Probes™) were obtained from Thermo Fisher Scientific. Cell nuclei were stained with TOTO®-3 solution (Thermo Fisher Scientific, 1:5000).

### Confocal microscopy and quantification of phenotypes

The samples were imaged using the confocal microscope TCS SP5II from Leica using 20× glycerol immersion objective. Settings were adjusted using the provided LAS AF software. Image data was processed and analyzed using ImageJ and Adobe Photoshop®. Line scan intensity profiles were generated using the “Plot profile” plugin in ImageJ by drawing a line across the lobe on a single representative confocal plane. Colocalization of Dscam[TM1] and FasII was analyzed using “Colocalisation threshold” plugin in ImageJ. Quantification of the axonal morphology and the Dscam[TM1]::GFP/Dscam[TM2]::GFP aggregates was done manually by analyzing the confocal Z-stacks and plotted as a percentage of MB where each brain was considered to have two individual MBs. For ORN targeting, the number of antennal lobes (two per brain) showing targeting defects were plotted as a percentage.

### Membrane fractionation

Detergent resistant membrane fractions were isolated as described before^70^ but with several modifications. Briefly, 200 *Drosophila* heads were lysed in 700 μl of TNET buffer (100 mM Tris pH 7.5, 150 mM NaCl, 2 mM EGTA, and 1% Triton X-100, 1X protease inhibitor) and then incubated on ice for 30 min. After incubation, the samples were spun at 1000 × *g* for 5 min at 4 °C and supernatant collected. The supernatant was then mixed with 60% Optiprep^TM^, overlaid with 30 and 5% Optiprep^TM^. The gradient was spun at 90,000 × *g* for 5 h at 4 °C using swinging bucket Thermo scientific Tft 80.4 rotor. Fractions were collected/numbered from the top and then probed using standard western blotting techniques. Rabbit anti-GFP (Invitrogen Life technologies A6455, 1:1000), mouse anti-syntaxin (DSHB 8C3, 1:100) and mouse anti- alpha-tubulin (DSHB 12G10, 1:200)

### Cloning and transgene production

Transgenic fly strains were created using the ɸC31 system^71^. *Spt-I* (RE58623) and *lace* (LD36009) cDNA was acquired from the *Drosophila* Genomics Resource Center (DGRC). TOPO cloning was performed using the pENTR™ Directional TOPO® Cloning Kit from Invitrogen. For the LR recombination reaction the Gateway® LR Clonase® II Enzyme mix from Invitrogen was used. The *pUASg-HA.attB* destination vector containing the Gateway® cassette, an UAS-site and a 3x hemagglutinin (HA) tag was kindly provided by the Konrad Basler lab, Zürich. The HA epitope tag was selected as previous studies showed that it does not impair SPT function^31,72^ and was fused to the C-terminal of the respective proteins for this study. Site-directed mutagenesis was performed using the QuikChange II XL Site-Directed Mutagenesis Kit from Agilent Technologies. Primers were designed using the QuikChange Primer Design program. The following primers were used for this study: *Spt-I* forward 5′-CAC CAT GGT GGC CAT CCA ATT G-3′; *Spt-I* reverse 5′-TAG GAC GGA GCT GGA AAC ACT CTC-3′; *Spt-I* ^*C129W*^ forward 5′-GAG TTG GAT CTT GGG GAC CTC GGG GCT-3′; *Spt-I* ^*C129W*^ reverse 5′-AGC CCC GAG GTC CCC AAG ATC CAA CTC-3′; *Spt-I* ^*C129Y*^ forward 5′-CGC AAG TAC GGA GTT GGA TCT TAT GGA CCT CGG GGC-3′; *Spt-I* ^*C129Y*^ reverse 5′-GCC CCG AGG TCC ATA AGA TCC AAC TCC GTA CTT GCG-3′; *Spt-I* ^*V140D*^ forward 5′-TAC GGC ACT ATG GAC GAT CAT CTG GAC CTG GAG G-3′; *Spt-I* ^*V140D*^ reverse 5′-CCT CCA GGT CCA GAT GAT CGT CCA TAG TGC CGT A-3′; *lace* forward 5′-CAC CAT GGG CAA TTT CGA CGG CG-3′; *lace* reverse 5′-GTA AAT GAC GGG ATT CGG ATC GCG-3′. All constructs were verified by sequencing before injection. Successful integration of the construct in the fly genome was verified by overexpression with *engrailed-Gal4* in the wing disc of *Drosophila* 3^rd^ instar larvae. The resulting expression pattern was visualized using anti-HA antibody. Transgene function was further verified by successful phenotypic rescue of *lace* ^*2*^*/lace* ^*k05*^ by overexpression of Lace^WT^.

### Sequencing of *Spt-I/lace* mutants

Different *Spt-I* and *lace* mutants were sequenced by isolating genomic DNA from a single fly using microLYSIS® Plus kit from microzone and then PCR amplifying using exon specific over-lapping primers designed using freeware Oligoanalyzer 3.1. The domain analysis of Spt-I/Lace was done using EMBL-Ebi InterPro^73^.

### Mass-spectrometric analysis

For Mass-spectrometric analysis, 20 male flies of each genotype 4-7 days post eclosion, were snap frozen in liquid nitrogen and then stored at −80^o^C until analysis. The experiment was done in 3 biological replicates.

Lipid extraction: The samples (10–16 mg) were weighed in 0.5 mL Precellys® CK14 Lysing Kit tubes (Bertin Instruments, Montigny-le-Bretonneux, France) and methanol was added (3 µL methanol/1 mg tissue). The samples were homogenized using a Precellys 24 tissue homogenizer (Bertin Instruments, Montigny-le-Bretonneux, France) equipped with a Cryolys cooling unit. Lipids were extracted using a modified methyl-tert-butyl ether (MTBE) method^74^. In brief, 20 µL of the sample homogenate were transferred into a glass vial, 10 µL internal standard solution and 120 µL methanol were added. After vortexing 500 µL MTBE were added and the mixture was incubated in a shaker for 10 min at room temperature. A phase separation was induced by adding 145 µL MS-grade water. After 10 min of incubation at room temperature, the samples were centrifuged at 1000 × *g* for 10 min. An aliquot of 450 µL of the upper phase (organic) was collected and dried in a vacuum concentrator. The samples were reconstituted in 200 µL methanol and used for LC-MS analysis.

Lipid standards: A mixture of deuterium labeled lipids (SPLASH® Lipidomix®) was purchased from Avanti Polar Lipids (Alabaster, AL, USA) and used as an internal standard.

Analysis of lipids using LC-MS: The LC-MS analysis was performed using a Vanquish UHPLC system (Thermo Fisher Scientific) combined with an Orbitrap Fusion™ Lumos™ Tribrid™ mass spectrometer (Thermo Fisher Scientific). Lipid separation was carried out by reversed phase chromatography employing an Accucore C18, 2.6 µm, 150 × 2 mm (Thermo Fisher Scientific) analytical column, column temperature was set to 35 °C. Acetonitrile/water (50/50, v/v) solution containing 10 mM ammonium formate and 0.1% formic acid was used as mobile phase A. Mobile phase B consisted of acetonitrile/isopropanol/water (10/88/2, v/v/v) containing 10 mM ammonium formate and 0.1% formic acid. The flow rate was set to 400 µL/min and a gradient of mobile phase B was applied to ensure optimal separation of the analyzed lipid species. The electrospray ionization in positive and negative mode was used for MS analysis, the MS conditions were as follows: capillary voltage, 3500 V (positive) and 3000 V (negative); vaporize temperature, 320 °C; ion transfer tube temperature, 285 °C; sheath gas, 60 arbitrary units; aux gas, 20 arbitrary units; sweep gas, 1 arbitrary unit. The Orbitrap MS scan mode at 120,000 mass resolution was employed for lipid detection. The scan range was set to 250–1200 m/z for both positive and negative ionization mode, the AGC target was set to 2.0e5 and the intensity threshold to 5.0e3. The data dependent MS2 scan using HCD with fixed collision energy mode set to 35% and inclusion list was employed to obtain MS2 spectra for Cer and Cer-PE lipid species. The data analysis was performed using the TraceFinder software (Thermo Fisher Scientific).

### Reporting summary

Further information on experimental design is available in the Nature Research Reporting Summary linked to this article.

## Supplementary information

Supplementary Information

Description of Additional Supplementary Files

Supplementary Data 1

Reporting Summary

## Data Availability

All mass-spectrometric data have been deposited to the EMBL-EMI MetaboLights database with the identifier MTBLS825^75^. All other data supporting the findings of this study are available within this manuscript and supplementary information files or from the authors upon request.
