# Supplementary material for: Sphingolipid-dependent Dscam sorting regulates axon segregation
Source: Nat Commun. 2019 Feb 18;10:813. doi: 10.1038/s41467-019-08765-2 (PMC6379420; doi:10.1038/s41467-019-08765-2)
Supplement: Supplementary file 1 — Supplementary Information [file 41467_2019_8765_MOESM1_ESM.pdf]

Sphingolipid-dependent Dscam sorting regulates axon segregation

Goyal G et al

A

Alleles	*Spt-I*^{P1}	*Spt-I*^{B2}	*Spt-I*^{49F}	*Spt-I*^{SH}	*Spt-I*^{KG}
Spt-I^{KG}	32.4 %	54.7 %	51.3 %	47.8 %	viable
Spt-I^{SH}	0.0 %	2.4 %	6.8 %	lethal	
Spt-I^{49F}	0.0 %	0.0 %	lethal		
Spt-I^{B2}	0.0 %	lethal			
Spt-I^{P1}	lethal				

Spt-I^{KG} < *Spt-I*^{SH} < *Spt-I*^{49F} < *Spt-I*^{B2} < *Spt-I*^{P1}

Alleles	*lace*^{2}	*lace*^{K05}	*lace*^{U2}	*lace*^{8}
lace^{8}	33.81%	31.92%	29.09%	lethal
lace^{U2}	0%	11.37%	lethal	
lace^{K05}	16.67%	lethal		
lace^{2}	lethal			

lace^{8} < *lace*^{k05} < *lace*^{2} < *lace*^{U2}

B

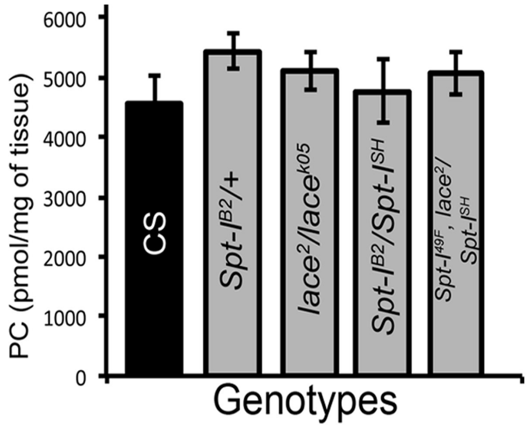

Supplementary Figure 1**: Complementation analysis and phospholipid levels in different *Spt-I* and *lace* alleles.**

(A) Complementation analysis of different *Spt-I* and *lace* alleles showing the percentage of adult flies viable as trans-heterozygotes with an allelic series showing the relative strength of the mutations.

(B) In adult flies, mass spectrometric analysis showed that levels of membrane phospholipid Phosphatidylcholine (PC) are not changed in *SPT* mutants. .Bars represent mean+/- SD across 3 biological replicates.

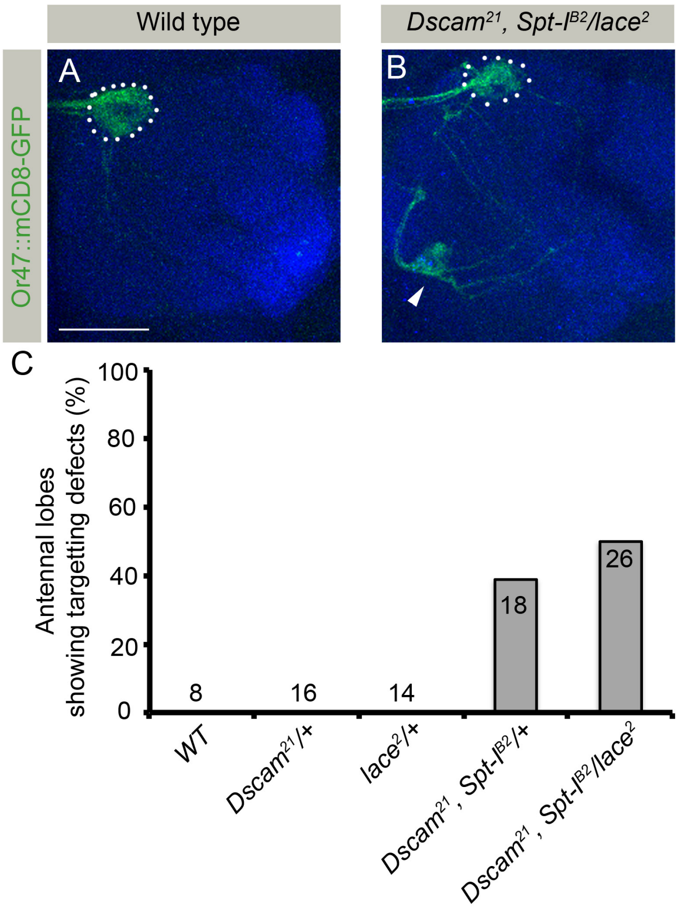

Supplementary Figure 2**: Genetic interaction between Dscam and *SPT* mutations during ORN targetting.**

[A,B] ORN-47a shows strong targeting defects in *Dscam*^{21},*Spt-I*^{B2}/*lace*^{2} (B) as compared to wild type (A). Scale Bar: 25µm

(C) Percentage of axonal lobes showing targeting defects in different genetic backgrounds. Numbers on the bars represent number of OL analyzed.

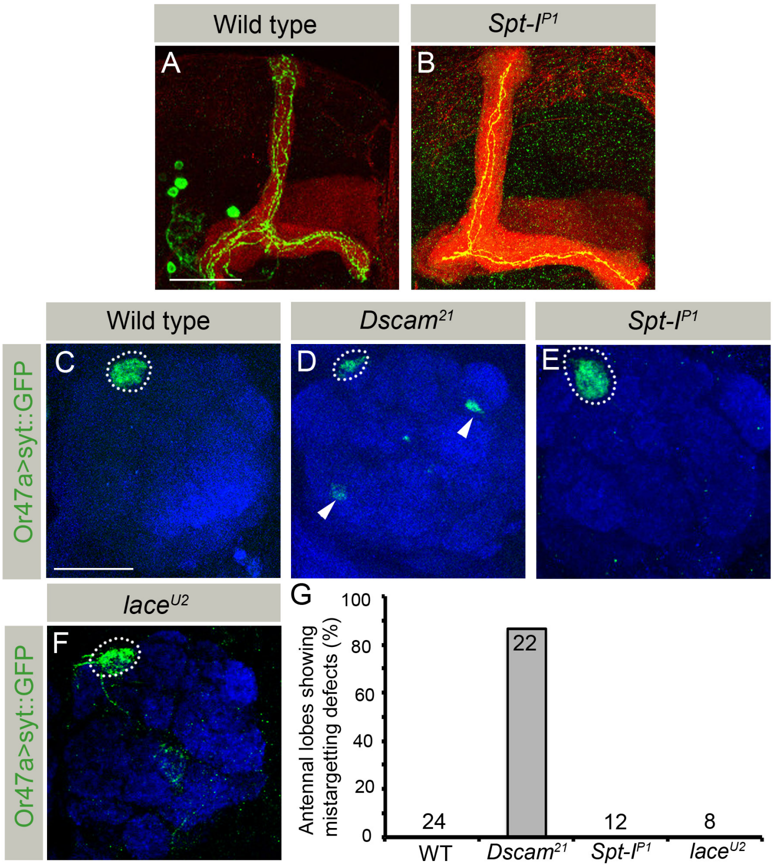

Supplementary Figure 3: ***SPT* mutants show no morphological phenotypes in small mosaic clones.**

[A,B] MARCM clones of wild type (A) and *Spt-I*^{P1} (B) in the MB of *Drosophila*. MARCM clones of *SPT-I*^{P1} do not show an axonal mistargeting phenotype.

[C-G] MARCM clones of Or47a>Syt::GFP in wild type (C), *Dscam*^{21} (D), *Spt-I*^{P1} (E) and *lace*^{U2} (F) background. MARCM clones of *Dscam*^{21} show ectopic targeting (D, arrowheads) as opposed to *Spt-I*^{P1} (E) and *lace*^{U2} (F) indicating a cell intrinsic function of Dscam and a cell-extrinsic rescue of the targeting defect in *SPT* mutants. (G) Percentage of antennal lobes showing targeting defects. Numbers on the bars represent number of OL analyzed. Scale Bar: 25µm

[A-F] Green: mCD8::GFP, Red: FasII, Blue: N-Cad.

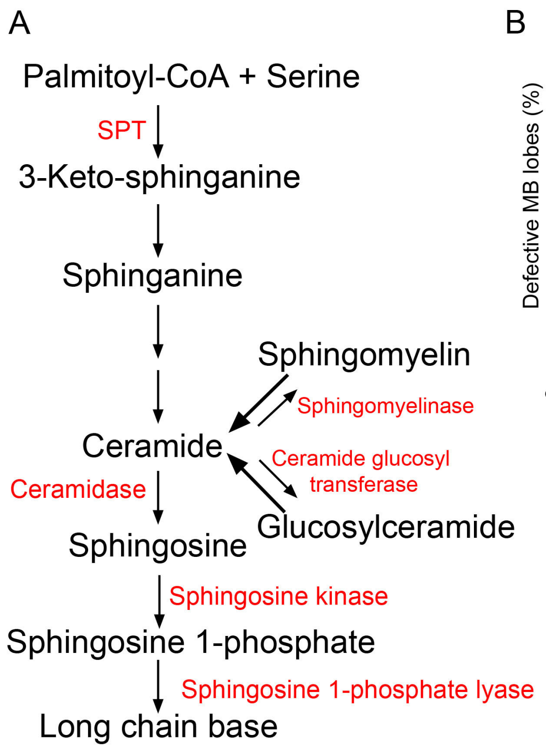

Supplementary Figure 4: **Knockdown of various enzymes catalyzing different steps of sphingolipid biosynthesis shows MB lost lobe phenotype.**

A) Schematic showing the multiple steps of sphingolipid biosynthesis pathway catalyzed by different enzymes and B) Percentage of MB lobes showing axonal defects on knock down of these enzymes using OK107-Gal4.

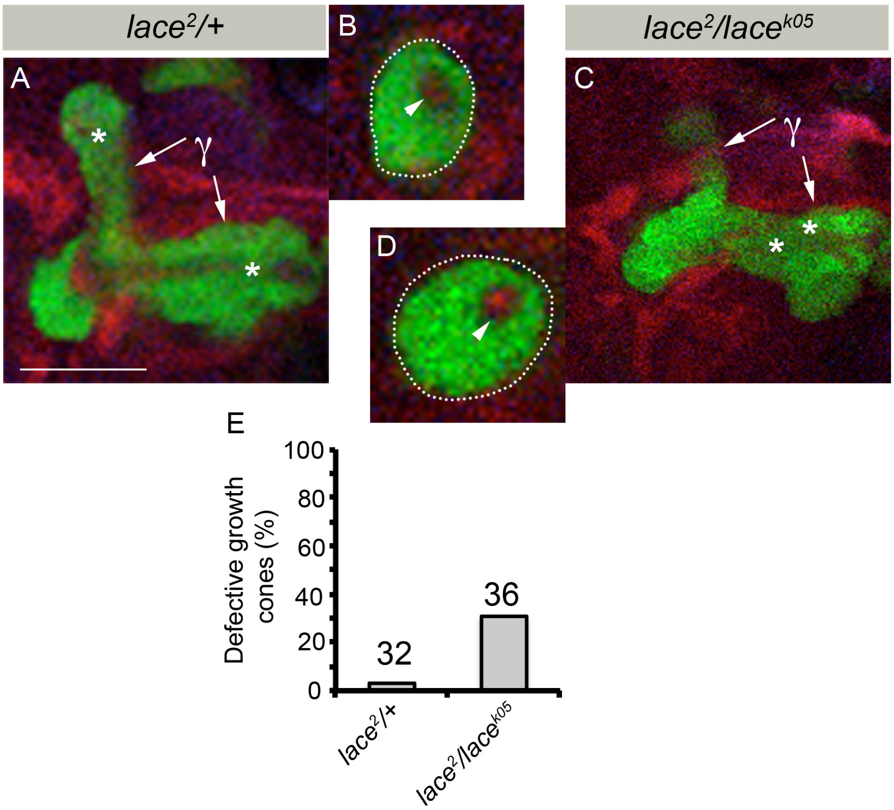

Supplementary Figure 5: ***SPT* mutants show normal proximal axon development while the distal axon shows morphological defects.**

[A-D] Third instar larval MB expressing mCD8::GFP (green) driven by 201Y-Gal4 in *lace²/+* (A,B) and *lace²/lace^k05^* (C,D) genetic backgrounds, co-stained with Flamingo (Red) to label growth cones. (B,D) represent cross-sections of peduncle in the respective genotypes. Reduced sphingolipids in *SPT* mutants cause non-segregation of the two growth cones (C, asterisks) although the development through the peduncle seems unperturbed (B and D, arrowheads). (E) Percentage of MB lobes showing non-segregation of growth cones. Numbers on the bars represent number of MB analyzed. Scale Bar: 25μm

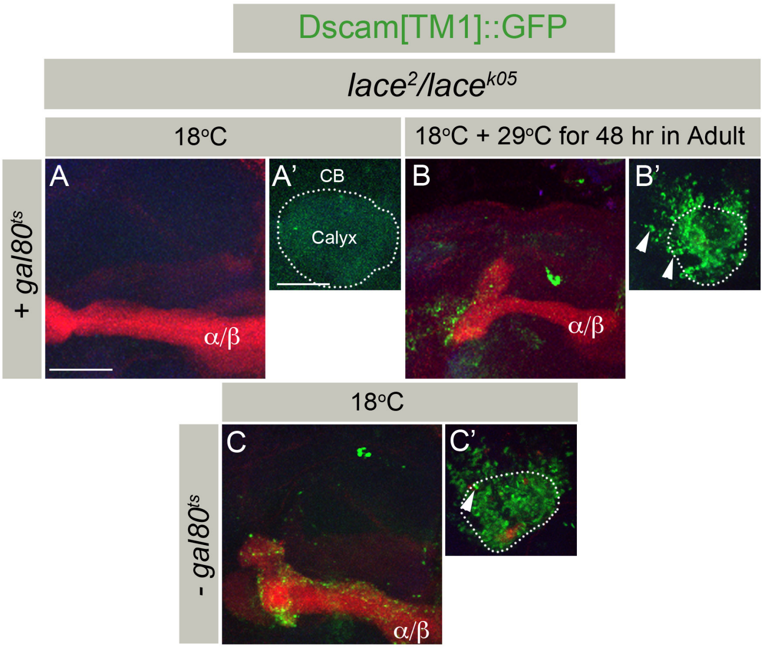

Supplementary Figure 6**: Sphingolipid depletion also affects mature neurons in the adult *Drosophila.***

[A-C] In *lace* mutants, expression of Dscam[TM1]::GFP using TARGET system (heat pulse for 48 hr in adult *Drosophila*) causes formation of aggregates (B', arrowheads). Even low level expression of Dscam[TM1]::GFP at 18ºC is sufficient to cause dendritic aggregation and axonal mislocalization (C). Scale Bar: 25μm Green: Dscam[TM1]::GFP, Red: FasII, Blue: N-Cad. n(A)=32, n(B)=6, n(C)=14

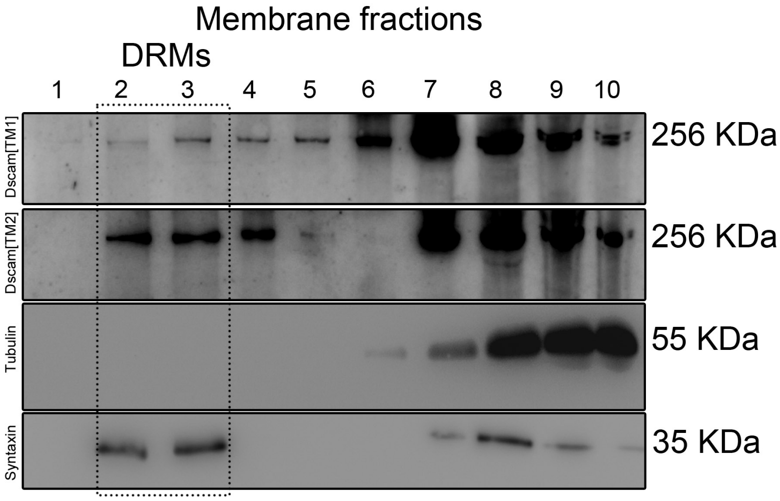

Supplementary Figure 7: **Dscam[TM2] is enriched in sphingolipid rich detergent resistant membrane (DRM) fraction.**

Representative western blots showing distribution of Dscam[TM1]/Dscam[TM2]/alpha-Tubulin and Syntaxin in *Drosophila* head membrane fractions. Dscam[TM1]::GFP and Dscam[TM2]::GFP were labeled using anti-GFP antibody. Syntaxin marks the sphingolipid rich[1] whereas alpha-tubulin marks sphingolipid low membrane fractions. The dotted box marks the DRM fraction. Genotypes: *DscamP-Dscam[TM1]::GFP* and *DscamP-Dscam[TM2]::GFP.*

Supplementary Methods

Different strains of *Drosophila*:

As wild type strain, Canton-S was used. Different transgenic lines used in the study were: *201Y-Gal4* [2], *OK107-Gal4* [3], *Dscam-Gal4* [4], *Act5C-Gal4* [5], *repo-Gal4* [6], *Elav*^{C155}*-Gal4* [7], *R16A06-Gal4* (γ neurons), *R26E01-Gal4* (α'/β' neurons), *R65G04-Gal4* (α/β neurons), *R30F11- Gal4* (α'/β' and early α/β neurons), *R26E01-lexA* (α'/β' neurons) (All Rubin collection driver lines [8]), *UAS-mCD8::GFP* [9], *UAS-Syt::GFP* [10], *UAS-Apc2::GFP* [11] ,*UAS-DenMark::cherry* [12], *UAS-nod::GFP* [13], *UAS-Vap33A* [14], *UAS-Dscam[TM1]::GFP* and *UAS-Dscam[TM2]::GFP* [4], *UAS-Dscam[TM1]::RFP* (Unpublished reagent from Prof. Tzumin Lee, Dscam transmembrane 1 isoform fused with RFP), *UAS-Dscam[TM1] RNAi* [15], *DscamP-Dscam[TM1]::GFP, DscamP-Dscam[TM2]::GFP* [4], *LexAop myr::cherry* [16], *Or47a>Syt::GFP, Or46a>Syt::GFP, Gr21a>CD2, Or47a::mCD8-GFP*. Different FasII domain variants were a kind gift from Prof. Akinao Nose [17].

To examine the function of the genes *Spt-I* and *lace,* several different alleles were selected for analysis. The alleles *Spt-I*^{KG06406}, *Spt-I*$^{l(2)49Fb4}$, *lace*^{2}, *lace*^{8} and *lace*^{k05305} were obtained from BDSC [18–22], *Spt-I*^{SH1626} was ordered from VDRC [23]. *Spt-I*^{B2}, *Spt-I*^{P1}, and *lace*^{U2} were identified during a mutagenesis screen done in our lab. The Dscam alleles: *Dscam*^{21}, *Dscam*^{24}, *Dscam*^{44} have been published before[24,25].

Supplementary Table 1: Exact genotypes of the flies

Figure	Genotypes
Figure 1D, WT	*eyflp/+; FRT42/FRT42 PCNA; Or47a>Syt::GFP*
Figure 1D, *SPT*	*eyflp/+; FRT42 Spt-I*^{P1}*/FRT42 PCNA; Or47a>Syt::GFP*
Figure 1D, *Dscam*	*eyflp/+; FRT42 Dscam*^{21}*/ FRT42 PCNA; Or47a>Syt::GFP*
Figure 1E, WT	*eyflp/+; FRT42 /FRT42 PCNA; Gr21a>CD2*
Figure 1E, *SPT*	*eyflp/+; FRT42 lace*^{U2}*/FRT42 PCNA; Gr21a>CD2*
Figure 1E, Dscam	*eyflp/+; FRT42 Dscam*^{21} */FRT42 PCNA; Gr21a>CD2*.
Figure 1U	*hsflp/+; FRT42 mCD8::GFP/FRT42 Gal80;; OK107-Gal4/+*
Figure 1V	*hsflp/+; FRT42 Dscam*^{21} *UAS-mCD8::GFP/FRT42 Gal80;; OK107-Gal4/+*
Figure 1W	*lace*^{2} *201Y-Gal4/+; UAS-FB1.1B/hsmflp5*
Figure 1X	*hsflp/+; lace*^{2} *201Y-Gal4/lace*^{k05}*; UAS>CD2>CD8::GFP/+*
Figure 2B	*R26E01-LexA/LexAop myr::cherry; R16A06-Gal4/UAS-mCD8::GFP*
Figure 2C	*lacek*^{05}*/+; R16A06-Gal4/UAS-mCD8::GFP*
Figure 2D	*lacek*^{05}*/+; R26E01-Gal4/UAS-mCD8::GFP*
Figure 2E	*lacek*^{05}*/+; R65G04-Gal4/UAS-mCD8::GFP*
Figure 2F	*lacek*^{05}*/lace*^{2}*; R16A06-Gal4/UAS-mCD8::GFP*
Figure 2G	*lacek*^{05}*/lace*^{2}*; R26E01-Gal4/UAS-mCD8::GFP*
Figure 2H	*lacek*^{05}*/lace*^{2}*; R65G04-Gal4/UAS-mCD8::GFP*
Figure 2J	*lace*^{2} *201Y-Gal4/+; UAS-mCD8::GFP/+*
Figure 2K-M	*lacek*^{05}*/+; R30F11-Gal4/UAS-mCD8::GFP*
Figure 2N	*lacek*^{05}*/+; UAS-mCD8::GFP/+; OK107-Gal4/+*
Figure 2O	*lace*^{2} *201Y-Gal4/lace*^{k05}*; UAS-mCD8::GFP/+*
Figure 2P-R	*lacek*^{05}*/lace*^{2}*; R30F11-Gal4/UAS-mCD8::GFP*
Figure 2S	*lacek*^{05}*/lace*^{2}*; UAS-mCD8::GFP/+; OK107-Gal4/+*
Figure 3A	*Spt-I*^{B2}*/+; UAS-Dscam[TM1]::GFP/+; OK107-Gal4/+*
Figure 3B	*Spt-I*^{B2}*/Spt-I*^{SH}*; UAS-Dscam[TM1]::GFP/+; OK107-Gal4/+*
Figure 3C	*Spt-I*^{49F}*, lace*^{U2}*/Spt-I*^{SH}*; UAS-Dscam[TM1]::GFP/+; OK107-Gal4/+*
Figure 3D	*lace*^{k05}*/+; UAS-Dscam[TM1]::GFP/+; OK107-Gal4/+*
Figure 3E	*lace*^{k05}*/lace*^{2}*; UAS-Dscam[TM1]::GFP/+; OK107-Gal4/+*
Figure 3F	*Spt-I*^{49F}*, lace*^{U2}*/lace*^{k05}*; UAS-Dscam[TM1]::GFP/+; OK107-Gal4/+*
Figure 3G	*lace*^{2} *Dscam-Gal4/lace*^{k05}*; UAS Dscam[TM1]::GFP/UAS-CD2*
Figure 3H	*lace*^{2} *Dscam-Gal4/lace*^{k05}*; UAS Dscam[TM1]::GFP/UAS-lace*^{WT}
Figure 3I	*lace2 201Y-Gal4/+; UAS-Dscam[TM2]::GFP/+*
Figure 3J	*lace2 201Y-Gal4/lace*^{k05}*; UAS-Dscam[TM2]::GFP/+*
Figure 3M	*DscamP-Dscam[TM1]::GFP/+; lace*^{k05}*/+*
Figure 3N	*DscamP-Dscam[TM1]::GFP/+; lace*^{2}*/lace*^{k05}
Figure 3O	*lace*^{k05}*/+; DscamP-Dscam[TM2]::GFP/+*
Figure 3P	*lace*^{2}*/lace*^{k05}*; DscamP-Dscam[TM2]::GFP/+*
Figure 4A	*lacek*^{05}*/+; R16A06-Gal4/UAS-Dscam[TM1]::GFP*

Figure 4B	*lacek^{05}/+; R26E01-Gal4/ UAS-Dscam[TM1]::GFP*
Figure 4C	*lacek^{05}/+; R65G04-Gal4/ UAS-Dscam[TM1]::GFP*
Figure 4D	*lacek^{05}/lace^{2}; R16A06-Gal4/ UAS-Dscam[TM1]::GFP*
Figure 4E	*lacek^{05}/lace^{2}; R26E01-Gal4/ UAS-Dscam[TM1]::GFP*
Figure 4F	*lacek^{05}/lace^{2}; R65G04-Gal4/ UAS-Dscam[TM1]::GFP*
Figure 4G	*lacek^{05}/+; R16A06-Gal4/ UAS-Dscam[TM2]::GFP*
Figure 4H	*lacek^{05}/+; R26E01-Gal4/ UAS-Dscam[TM2]::GFP*
Figure 4I	*lacek^{05}/+; R65G04-Gal4/ UAS-Dscam[TM2]::GFP*
Figure 4J	*lacek^{05}/lace^{2}; R16A06-Gal4/ UAS-Dscam[TM2]::GFP*
Figure 4K	*lacek^{05}/lace^{2}; R26E01-Gal4/ UAS-Dscam[TM2]::GFP*
Figure 4L	*lacek^{05}/lace^{2}; R65G04-Gal4/ UAS-Dscam[TM2]::GFP*
Figure 4M	*lacek^{05}/+; R65G04-Gal4/ UAS-Dscam[TM1]::GFP*
Figure 4N	*lacek^{05}/+; R65G04-Gal4/ UAS-Dscam[TM2]::GFP*
Figure 4O	*lacek^{05}/lace^{2}; R65G04-Gal4/ UAS-Dscam[TM1]::GFP*
Figure 4P	*lacek^{05}/lace^{2}; R65G04-Gal4/ UAS-Dscam[TM2]::GFP*
Figure 5A	*lace^{k05}/+; UAS-Apc2::GFP/+; OK107-Gal4/+*
Figure 5B	*lace^{k05}/+; UAS-nod::GFP/+; OK107-Gal4/+*
Figure 5C	*lace^{k05}/+; UAS-Syt::GFP, UAS-DenMark::cherry/+; OK107-Gal4/+*
Figure 5D	*lace^{k05}/lace^{2}; UAS-Apc2::GFP/+; OK107-Gal4/+*
Figure 5E	*lace^{k05}/ lace^{2}; UAS-nod::GFP/+; OK107-Gal4/+*
Figure 5F	*lace^{k05}/ lace^{2}; UAS-Syt::GFP, UAS-DenMark::cherry/+; OK107-Gal4/+*
Figure 5G	*Spt-I^{49F}, lace^{2}/+*
Figure 5H	*Spt-I^{49F}, lace^{2}/Spt-I^{SH}*
Figure 5I	*Spt-I^{49F}, lace^{2}/lace^{k05}*
Figure 5J, M	*Spt-I^{49F}, lace^{2}/+; UAS-Dscam[TM1]::GFP/+; OK107-Gal4/+*
Figure 5K, N	*Spt-I^{49F}, lace^{2}/Spt-I^{SH}; UAS-Dscam[TM1]::GFP/+; OK107-Gal4/+*
Figure 5L, O	*Spt-I^{49F}, lace^{2}/lace^{k05}; UAS-Dscam[TM1]::GFP/+; OK107-Gal4/+*
Figure 5P	*lace^{2} 201Y-Gal4/+; UAS-Dscam[TM1]::GFP*
Figure 5Q	*lace^{2} 201Y-Gal4/+; UAS-Dscam[TM2]::GFP*
Figure 5R	*lace^{2} 201Y-Gal4/+; UAS-Apc2::GFP*
Figure 5S	*lace^{2} 201Y-Gal4/+; UAS-Syt::GFP*
Figure 5T	*lace^{2} 201Y-Gal4/lace^{k05}; UAS-Dscam[TM1]::GFP*
Figure 5U	*lace^{2} 201Y-Gal4/ lace^{k05}; UAS-Dscam[TM2]::GFP*
Figure 5V	*lace^{2} 201Y-Gal4/ lace^{k05}; UAS-Apc2::GFP*
Figure 5W	*lace^{2} 201Y-Gal4/ lace^{k05}; UAS-Syt::GFP*
Figure 6A	*lace^{k05}/+; UAS-mCD8::GFP/+; OK107-Gal4/+*
Figure 6B	*lace^{k05}/+; UAS-Dscam[TM1]::GFP/+; OK107-Gal4/+*
Figure 6C	*lace^{k05}/+; UAS-Apc2::GFP/+; OK107-Gal4/+*
Figure 6D	*lace^{k05}/lace^{2}; UAS-mCD8::GFP/+; OK107-Gal4/+*
Figure 6E	*lace^{k05}/ lace^{2}; UAS-Dscam[TM1]::GFP/+; OK107-Gal4/+*
Figure 6F	*lace^{k05}/ lace^{2}; UAS-Apc2::GFP/+; OK107-Gal4/+*
Figure 6G	*R26E01-Gal4/UAS-Dscam[TM1]::GFP*
Figure 6H	*UAS-FasII^{PEST-}/+; R26E01-Gal4/+*
Figure 6I	*UAS-FasII^{PEST-}/+; R26E01-Gal4/ UAS-Dscam[TM1]::GFP*
Figure 6J	*UAS-FasII^{PEST-}/+; R26E01-Gal4/ UAS-Dscam[TM1] RNAi*

Figure 6K	*UAS-Dscam[TM1]::RFP/+; R26E01-Gal4/ UAS-FasII^{intra}::YFP*
Figure 6L	*UAS-Dscam[TM1]::RFP/+; R26E01-Gal4/ UAS-FasII^{extra}::YFP*
Figure 7B	*lace2 201Y-Gal4/+; UAS-Dscam[TM2]::GFP/+*
Figure 7C	*lace2 201Y-Gal4/lace^{k05}; UAS-Dscam[TM2]::GFP/+*
Figure 7D	*UAS-Dscam[TM1]::RFP/+; R65G04-Gal4/+*
Figure 7E	*UAS-Dscam[TM2]::GFP/ R65G04-Gal4*
Figure 7F	*UAS-Dscam[TM1]::RFP/+; R65G04-Gal4/ UAS-Dscam[TM2]::GFP*
Figure 7G, I	*lace^{k05} UAS-Dscam[TM1]::RFP/+; R65G04-Gal4/ UAS-Dscam[TM2]::GFP*
Figure 7H, J	*lace^{k05} UAS-Dscam[TM1]::RFP/lace^{2}; R65G04-Gal4/ UAS-Dscam[TM2]::GFP*
Figure 8A, B	*lace^{k05}/+; R65G04-Gal4/ UAS-Dscam[TM1]::GFP*
Figure 8C, D	*lace^{k05}/lace^{2}; R65G04-Gal4/ UAS-Dscam[TM1]::GFP*
Figure 8E, F	*lace^{k05}/+; R65G04-Gal4/ UAS-Dscam[TM2]::GFP*
Figure 8G, H	*lace^{k05}/lace^{2}; R65G04-Gal4/ UAS-Dscam[TM2]::GFP*
Figure 9A-C	*lace^{k05}/+; R30F11-Gal4/Dscam[TM2]::GFP*
Figure 9D-F	*lace^{k05}/lace^{2}; R30F11-Gal4/Dscam[TM2]::GFP*
Figure 9G, I	*lace^{2} 201Y-Gal4/+; UAS-mCD8::GFP/+*
Figure 9H, J	*lace^{2} 201Y-Gal4/+; UAS-Dscam[TM2]::GFP/+*
Figure 9K, M	*lace^{2} 201Y-Gal4/+; UAS-Dscam[TM2]::GFP/+*
Figure 9L, N	*lace^{2} 201Y-Gal4/lace^{k05}; UAS-Dscam[TM2]::GFP/+*
Figure 10A	*Act5C-Gal4/+; UAS-Spt-I^{WT}*
Figure 10B	*Act5C-Gal4/+; UAS-Spt-I^{C129W}*
Figure 10C	*Act5C-Gal4/+; UAS-Spt-I^{C129Y}*
Figure 10D	*Act5C-Gal4/+; UAS-Spt-I^{V140D}*
Figure 10E, I	*UAS-Spt-I^{WT}/UAS-Dscam[TM1]::GFP; OK107-Gal4/+*
Figure 10F, J	*UAS-Spt-I^{C129W}/UAS-Dscam[TM1]::GFP; OK107-Gal4/+*
Figure 10G, K	*UAS-Spt-I^{C129Y}/UAS-Dscam[TM1]::GFP; OK107-Gal4/+*
Figure 10H, L	*UAS-Spt-I^{V140D}/UAS-Dscam[TM1]::GFP; OK107-Gal4/+*
Supp. Fig. 2A	*Or47a::mCD8::GFP/+*
Supp. Fig. 2B	*Dscam^{21}, Spt-I^{B2}/lace^{2}; Or47a::mCD8::GFP/+*
Supp. Fig. 3A	*hsflp/+; FRT42 mCD8::GFP/FRT42 Gal80;; OK107 gal4/+*
Supp. Fig. 3B	*hsflp/+; FRT42 Spt-I^{P1} mCD8::GFP/FRT42 Gal80;; OK107 gal4/+*
Supp. Fig. 3C	*eyflp/+; FRT42 /FRT42 Gal80; Or47a>Syt::GFP*
Supp. Fig. 3D	*eyflp/+; FRT42 Dscam^{21}/FRT42 Gal80; Or47a>Syt::GFP/+*
Supp. Fig. 3E	*eyflp/+; FRT42 Spt-I^{P1}/FRT42 Gal80; Or47a>Syt::GFP/+*
Supp. Fig. 3F	*eyflp/+; FRT40 lace^{U2}/FRT40 Gal80; Or47a>Syt::GFP/+*
Supp. Fig. 5A	*lace^{2} 201Y-Gal4/+; UAS-mCD8::GFP/+*
Supp. Fig. 5B	*lace^{2} 201Y-Gal4/lace^{k05}; UAS-mCD8::GFP/+*
Supp. Fig. 6A,B	*lace^{2} Dscam-Gal4/lace^{k05}; UAS Dscam[TM1]::GFP/Gal80^{ts}*
Supp. Fig. 6C	*lace^{2} Dscam-Gal4/lace^{k05}; UAS Dscam[TM1]::GFP/+*

Supplementary References:

1. Fernandez-Funez, P. *et al.* In vivo generation of neurotoxic prion protein: Role for Hsp70 in accumulation of misfolded isoforms. *PLoS Genet.* **5,** (2009).
2. Yang, M. Y., Armstrong, J. D., Vilinsky, I., Strausfeld, N. J. & Kaiser, K. Subdivision of the Drosophila mushroom bodies by enhancer-trap expression patterns. *Neuron* **15,** 45–54 (1995).
3. Connolly, J. B. *et al.* Associative learning disrupted by impaired Gs signaling in Drosophila mushroom bodies. *Science* **274,** 2104–7 (1996).
4. Wang, J. *et al.* Transmembrane/juxtamembrane domain-dependent Dscam distribution and function during mushroom body neuronal morphogenesis. *Neuron* **43,** 663–72 (2004).
5. Ito, K., Awano, W., Suzuki, K., Hiromi, Y. & Yamamoto, D. The Drosophila mushroom body is a quadruple structure of clonal units each of which contains a virtually identical set of neurones and glial cells. *Development* **124,** 761–71 (1997).
6. Lee, B. P. & Jones, B. W. Transcriptional regulation of the Drosophila glial gene repo. *Mech. Dev.* **122,** 849–862 (2005).
7. Lin, D. M. & Goodman, C. S. Ectopic and increased expression of Fasciclin II alters motoneuron growth cone guidance. *Neuron* **13,** 507–523 (1994).
8. Jenett, A. *et al.* A GAL4-Driver Line Resource for Drosophila Neurobiology. *Cell Rep.* **2,** 991–1001 (2012).
9. Lee, T. & Luo, L. Mosaic analysis with a repressible cell marker for studies of gene function in neuronal morphogenesis. *Neuron* **22,** 451–61 (1999).
10. Zhang, Y. Q., Rodesch, C. K. & Broadie, K. Living synaptic vesicle marker: synaptotagmin-GFP. *Genesis* **34,** 142–145 (2002).
11. Akong, K., McCartney, B. M. & Peifer, M. Drosophila APC2 and APC1 have overlapping roles in the larval brain despite their distinct intracellular localizations. *Dev. Biol.* **250,** 71–90 (2002).
12. Nicolai, L. J. J. *et al.* Genetically encoded dendritic marker sheds light on neuronal connectivity in Drosophila. *Proc. Natl. Acad. Sci.* **107,** 20553–20558 (2010).
13. Andersen, R., Li, Y., Resseguie, M. & Brenman, J. E. Calcium/calmodulin-dependent protein kinase II alters structural plasticity and cytoskeletal dynamics in Drosophila. *J. Neurosci.* **25,** 8878–88 (2005).
14. Yang, Z. *et al.* Drosophila Vap-33 is required for axonal localization of Dscam isoforms. *J. Neurosci.* **32,** 17241–50 (2012).
15. Shi, L., Yu, H.-H., Yang, J. S. & Lee, T. Specific *Drosophila* Dscam juxtamembrane variants control dendritic elaboration and axonal arborization. *J. Neurosci.* **27,** 6723–6728 (2007).
16. Diegelmann, S., Bate, M. & Landgraf, M. Gateway cloning vectors for the LexA-based binary expression system in drosophila. *Fly (Austin).* **2,** 236–239 (2008).
17. Kohsaka, H., Takasu, E. & Nose, A. In vivo induction of postsynaptic molecular assembly by the cell adhesion molecule Fasciclin2. *J. Cell Biol.* **179,** 1289–1300 (2007).
18. Bellen, H. J. *et al.* The BDGP gene disruption project: single transposon

insertions associated with 40% of Drosophila genes. *Genetics* **167,** 761–81 (2004).

19. Lasko, P. F. & Pardue, M. Lou. Studies of the genetic organization of the vestigial microregion of Drosophila melanogaster. *Genetics* **120,** 495–502 (1988).
20. Ashburner, M., Detwiler, C., Tsubota, S. & Woodruff, R. C. The genetics of a small autosomal region of Drosophila melanogaster containing the structural gene for alcohol dehydrogenase. VI. Induced revertants of scutoid. *Genetics* **104,** 405–31 (1983).
21. Spradling, A. C. *et al.* The Berkeley Drosophila Genome Project gene disruption project: Single P-element insertions mutating 25% of vital Drosophila genes. *Genetics* **153,** 135–77 (1999).
22. Ashburner, M. *et al.* The genetics of a small autosomal region of Drosophila melanogaster containing the structural gene for alcohol dehydrogenase. VII. Characterization of the region around the snail and cactus loci. *Genetics* **126,** 679–694 (1990).
23. Oh, S. W. *et al.* A P-element insertion screen identified mutations in 455 novel essential genes in Drosophila. *Genetics* **163,** 195–201 (2003).
24. Hummel, T. *et al.* Axonal targeting of olfactory receptor neurons in Drosophila is controlled by Dscam. *Neuron* **37,** 221–31 (2003).
25. Zhan, X.-L. L. *et al.* Analysis of Dscam diversity in regulating axon guidance in Drosophila mushroom bodies. *Neuron* **43,** 673–86 (2004).
