# Supplementary material for: Sphingolipid-dependent Dscam sorting regulates axon segregation
Source: Nat Commun. 2019 Feb 18;10:813. doi: 10.1038/s41467-019-08765-2 (PMC6379420; doi:10.1038/s41467-019-08765-2)
Supplement: Supplementary file 2 — Description of Additional Supplementary Files [file 41467_2019_8765_MOESM2_ESM.pdf]

Description of Additional Supplementary Information

File Name: Supplementary Dataset 1

Description: Raw data of mass spectrometry analysis for measurement of lipids in SPT mutants. The file contains raw data of mass spectrometry analysis for different Ceramide, Ceramide Phosphoethanolamine (CerPE) and Phosphatidyl Choline (PC) species detected and analyzed in 3 biological replicates of different genetic combinations. The numbers represent pmol/mg of tissue.
